# Omega-3 fatty acid blood levels are inversely associated with cardiometabolic risk factors in HFpEF patients: the Aldo-DHF randomized controlled trial

**DOI:** 10.1007/s00392-021-01925-9

**Published:** 2021-08-28

**Authors:** Katharina Lechner, Johannes Scherr, Elke Lorenz, Benjamin Lechner, Bernhard Haller, Alexander Krannich, Martin Halle, Rolf Wachter, André Duvinage, Frank Edelmann

**Affiliations:** 1 Department of Prevention, Rehabilitation and Sports Medicine, School of Medicine, Technical University of Munich, Munich, Germany; 2 DZHK (German Centre for Cardiovascular Research), Partner site Munich, Munich Heart Alliance, Munich, Germany; 3 Kardiologie, Deutsches Herzzentrum München, Munich, Germany; 4 University Center for Prevention and Sports Medicine, Balgrist University Hospital, University of Zurich, Zürich, Switzerland; 5 Department of Internal Medicine IV, Ludwig-Maximilians University, Munich, Germany; 6 Institute of Medical Informatics, Statistics and Epidemiology, Klinikum rechts der Isar, Technische Universität München, Munich, Germany; 7 Charité, Universitätsmedizin Berlin, Berlin, Germany; 8 Clinic and Policlinic for Cardiology, University Hospital Leipzig, Leipzig, Germany; 9 Department of Cardiology and Pneumology, Georg-August University, University Medical Center Göttingen, Göttingen, Germany; 10 Department of Cardiology, Campus Virchow Klinikum (CVK), Charité, Universitätsmedizin Berlin, Augustenburger Platz 1, 13353 Berlin, Germany

**Keywords:** Omega-3 fatty acids, Omega-3 index, Eicosapentaenoic acid, Docosahexaenoic acid, Heart failure, HFpEF, Diastolic dysfunction, Metabolic phenotype, Atherogenic dyslipidemia, Functional capacity

## Abstract

**Objectives:**

To evaluate associations of omega-3 fatty acid (O3-FA) blood levels with cardiometabolic risk markers, functional capacity and cardiac function/morphology in patients with heart failure with preserved ejection fraction (HFpEF).

**Background:**

O3-FA have been linked to reduced risk for HF and associated phenotypic traits in experimental/clinical studies.

**Methods:**

This is a cross-sectional analysis of data from the Aldo-DHF-RCT. From 422 patients, the omega-3-index (O3I = EPA + DHA) was analyzed at baseline in *n* = 404 using the HS-Omega-3-Index^®^ methodology. Patient characteristics were; 67 ± 8 years, 53% female, NYHA II/III (87/13%), ejection fraction ≥ 50%, *E*/*e*′ 7.1 ± 1.5; median NT-proBNP 158 ng/L (IQR 82–298). Pearson’s correlation coefficient and multiple linear regression analyses, using sex and age as covariates, were used to describe associations of the O3I with metabolic phenotype, functional capacity, echocardiographic markers for LVDF, and neurohumoral activation at baseline/12 months.

**Results:**

The O3I was below (< 8%), within (8–11%), and higher (> 11%) than the target range in 374 (93%), 29 (7%), and 1 (0.2%) patients, respectively. Mean O3I was 5.7 ± 1.7%. The O3I was inversely associated with HbA1c (*r* = − 0.139, *p* = 0.006), triglycerides-to-HDL-C ratio (*r* = − 0.12, *p* = 0.017), triglycerides (*r* = − 0.117, *p* = 0.02), non-HDL-C (*r* = − 0.101, *p* = 0.044), body-mass-index (*r* = − 0.149, *p* = 0.003), waist circumference (*r* = − 0.121, *p* = 0.015), waist-to-height ratio (*r* = − 0.141, *p* = 0.005), and positively associated with submaximal aerobic capacity (*r* = 0.113, *p* = 0.023) and LVEF (*r* = 0.211, *p* < 0.001) at baseline. Higher O3I at baseline was predictive of submaximal aerobic capacity (*β* = 15.614, *p* < 0,001), maximal aerobic capacity (*β* = 0.399, *p* = 0.005) and LVEF (*β* = 0.698, *p* = 0.007) at 12 months.

**Conclusions:**

Higher O3I was associated with a more favorable cardiometabolic risk profile and predictive of higher submaximal/maximal aerobic capacity and lower BMI/truncal adiposity in HFpEF patients.

**Graphic abstract:**

Omega-3 fatty acid blood levels are inversely associated with cardiometabolic risk factors in HFpEF patients. Higher O3I was associated with a more favorable cardiometabolic risk profile and aerobic capacity (left) but did not correlate with echocardiographic markers for left ventricular diastolic function or neurohumoral activation (right). An O3I-driven intervention trial might be warranted to answer the question whether O3-FA in therapeutic doses (with the target O3I 8–11%) impact on echocardiographic markers for left ventricular diastolic function and neurohumoral activation in patients with HFpEF. This figure contains modified images from Servier Medical Art (https://smart.servier.com) licensed by a Creative Commons Attribution 3.0 Unported License.

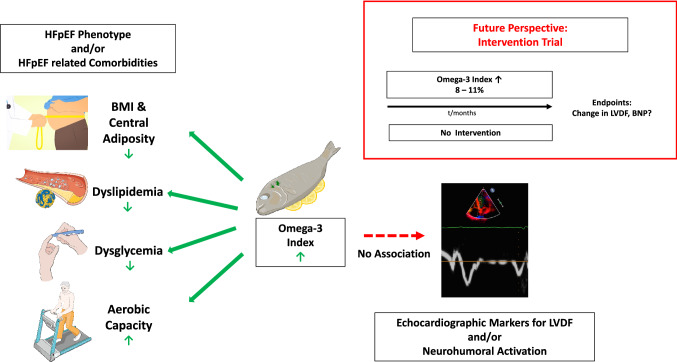

**Supplementary Information:**

The online version contains supplementary material available at 10.1007/s00392-021-01925-9.

## Introduction

The prevalence of heart failure (HF) with preserved ejection fraction (HFpEF) relative to heart failure with reduced ejection fraction (HFrEF) continues to increase [1]. HFpEF has similar mortality to HFrEF, however, standard pharmacological therapies as applied in HFrEF have failed to show prognostic impact in HFpEF [2, 3]. The rising prevalence of HFpEF and the absence of established pharmacological treatment options result in individual suffering and socioeconomic burden [3].

HFpEF clinically presents as a heterogeneous condition with numerous underlying aetiologies [4] but conceptually relates to a distinct metabolic phenotype characterized by extracardiac comorbidities such as obesity, arterial hypertension (HT), and type 2 diabetes mellitus (T2D) [1]. A unifying hallmark of these metabolic features is the accumulation of ectopic adipose tissue in the epicardium [5]. The latter drives left ventricular remodeling and dysfunction through altered paracrine signaling to cardiomyocytes, through macrophage infiltration, and interstitial fibrosis and through systemic inflammation and coronary microvascular endothelial dysfunction [1, 2].

The two long-chain omega-3 fatty acids (O3-FA) eicosapentaenoic acid (EPA; C20:5n3) and/or docosahexaenoic acid (DHA; C22:6n3) have been shown to reduce blood pressure, biomarkers of myocardial fibrosis such as ST2, ameliorate left ventricular diastolic function, and decrease markers of natriuretic peptide system activation and wall stress like brain natriuretic peptide (BNP) in HF patients [6–8]. In line, higher baseline plasma levels of EPA were significantly associated with reduced risk for HFrEF and HFpEF in the Multi-Ethnic Study of Atherosclerosis (MESA) [9]. Biologic properties of EPA&DHA provide plausibility for this observation; they are anti-inflammatory [10, 11], improve vascular endothelial function [12], decrease systolic/diastolic blood pressure [13], decrease resting heart rate [13], increase heart rate variability [14, 15], have anti-arrhythmic properties [16], and decrease cardiac remodeling [7]. In part mediated by these effects, O3-FA may impact on cardiomeabolic phenotype in HFpEF [17]. In HFrEF, O3-FA have been endorsed as a safe treatment option on top of guideline-based usual care with a Class IIa and Class IIb recommendation by the AHA [18] and ESC [2] respectively.

As measured, the effects of EPA&DHA correlate with the omega-3 index (O3I), a biomarker for tissue O3-FA status [7, 19] which is unbiased by recent dietary intake and reliably reflects long-term cardiac and other tissue EPA&DHA levels in the absence and presence of supplementation [20, 21]. The O3I tracks more closely with clinical endpoints than dietary intake, as has been consistently demonstrated in intervention and observational studies [7, 22, 23].

The association between EPA&DHA tissue levels and patient characteristics in HFpEF is not known. To fill this gap, we report the O3I in a large cohort comprised of 404 HFpEF patients from the Aldosterone in Diastolic Heart Failure (Aldo-DHF) trial and associations with metabolic phenotype, functional capacity, echocardiographic markers indicative of left ventricular diastolic function (LVDF), and neurohumoral activation. We hypothesized that the O3I would correlate inversely with biomarkers indicative of cardiometabolic risk, LVDF, and neurohumoral activation in patients with HFpEF.

## Methods

### Study design

This is a post-hoc analysis of the Aldo-DHF trial (ISRCTN 94726526). We analyzed O3-FA blood cell membrane levels at baseline. From a total of 422 patients enrolled in the Aldo-DHF trial, 18 whole blood aliquots were not available due to loss during storage/transfer or missing blood sampling at baseline. Baseline characteristics are depicted in Table 1.

**Table 1:** Baseline characteristics

Characteristics^a^	Total (*n* = 404)
Demographics	
Age, mean (SD), y	67 (8)
Female	212 (53)
Laboratory measures	
HbA1c (%)	6.0 (0.8)
LDL-C (mg/dl)	117 (42)
HDL-C (mg/dl)	56 (18)
Triglycerides (mg/dl)	161 (103)
Non-HDL-C (mg/dl)	133 (47)
TG/HDL-C ratio	3.3 (2.8)
NT-proBNP, median (IQR), ng/L	158 (82–298)
Omega-3 Index (%)	5.7 (1.7)
Medical history	
Hospitalization for heart failure in past 12 months^c^	149 (37)
Hypertension	370 (92)
Diabetes mellitus	66 (16)
Atrial fibrillation	65 (16)
Physical examination, mean (SD)	
Body mass index^b^	28.9 (3.6)
Waist circumference, (cm) In men In women	98.1 (11.0) 103.7 (9.0) 93.1 (10.3)
Waist-to-height ratio	0.49 (0.1)
Systolic blood pressure, mm Hg	135 (18)
Diastolic blood pressure, mm Hg	79 (11)
Heart rate, /min	66 (11)
Signs and symptoms	
NYHA functional class	
II	350 (87)
III	54 (13)
Peripheral edema	160 (40)
Nocturia	325 (80)
Paroxysmal nocturnal dyspnea	66 (16)
Nocturnal cough	61 (15)
Fatigue	241 (60)
Current medications	
ACE inhibitors/angiotensin receptor antagonists	310 (77)
Betablockers	290 (72)
Diuretics	213 (53)
Calcium antagonists	97 (24)
Lipid-lowering drugs	221 (55)
Echocardiography, mean (SD)	
LV ejection fraction, %	68 (8)
LV diameter (end diastolic), mm	46.5 (6.2)
LV diameter (end systolic), mm	25.3 (6.4)
LV-mass index, g/m^2^	114.15 (45.53)
Left atrial volume index, mL/m^2^	43.1 (41.6)
*E*-wave velocity, cm/s	73 (20)
Medial *e*′ wave velocity, cm/s	5.9 (1.3)
*E*/*e*′	7.1 (1.5)
*E*/*A* velocity ratio	0.91 (0.33)
Isovolumic relaxation time, ms	88 (26)
Deceleration time, ms	243 (63)
Grade of diastolic dysfunction, no. (%)	
I	295 (73)
II	81 (20)
III	4 (1)
IV	3 (1)

*ACE* angiotensin-converting enzyme, *IQR* interquartile range, *NT-proBNP* N-terminal pro–brain-type natriuretic peptide, *NYHA* New York Heart Association, *A* peak atrial transmitral ventricular filling velocity, *e'* early diastolic tissue Doppler velocity, *E* peak early transmitral ventricular filling velocity

^a^Data are expressed as No. (%) unless otherwise specified

^b^Body mass index is defined as weight in kilograms divided by height in meters squared

^c^2 missing from analysis (*n* = 402)

### The Aldo-DHF trial

The Aldo-DHF trial is a multicenter, prospective, randomized, double-blind, placebo-controlled trial evaluating the effect of a 12 months aldosterone receptor blockade on diastolic function (*E*/*e*′) on echocardiography and maximal exercise capacity (VO2peak) in HFpEF patients. Men and women aged 50 years or older were eligible to participate in the study if they had current HF symptoms consistent with New York Heart Association (NYHA) class II or III, left ventricular ejection fraction (LVEF) of 50% or greater, echocardiographic evidence of diastolic dysfunction (grade I) or atrial fibrillation at presentation, and maximum exercise capacity (VO2peak) of 25 mL/kg/min or less [24]. 422 ambulatory patients (mean age 67 [SD, 8] years; 52% female) with chronic NYHA class II or III heart failure, preserved left ventricular ejection fraction (LVEF) of 50% or greater, and evidence of diastolic dysfunction were included. Data acquisition was conducted between March 2007 and April 2012 at 10 sites in Germany and Austria [24].

### Clinical measurements

#### Laboratory methods: Aldo-DHF trial

Venous blood samples were drawn under standardized conditions after 20 min of rest in supine position. Samples were immediately cooled, centrifuged, and processed for storage at − 80 °C (− 112 °F). N-terminal pro–brain-type natriuretic peptide (NT-proBNP) was analyzed with the Elecsys NT-proBNP immunoassay (Roche Diagnostics) [24].

#### Laboratory methods: HS-Omega-3 Index^®^ methodology

Blood samples from the Aldo-DHF trial were immediately stored at − 80 °C. This results in stable O3 levels [25]. For analysis of fatty acid composition, 2.0 mL aliquots of frozen (− 80 °C) EDTA-blood were sent to Omegametrix (Martinsried, Germany). At Omegametrix, a reference laboratory for fatty acid analyses, whole blood fatty acid composition was analyzed according to the HS-Omega-3 Index^®^ methodology [26]. Fatty acid methyl esters were generated by acid transesterification and were analyzed by gas chromatography using a GC2010 Gas Chromatograph (Shimadzu, Duisburg, Germany) equipped with a SP2560, 100-m column (Supelco, Bellefonte, Pennsylvania, United States) using hydrogen as carrier gas. Fatty acids were identified by comparison with a standard mixture of fatty acids characteristic of erythrocytes. Results for EPA&DHA from whole blood aliquots were recalculated into the erythrocyte O3I by the use of a validated correction factor, [26] while individual fatty acid results are given as relative amounts of ALA (C18:3n3), EPA (C20:5n3) and DHA (C22:6n3) expressed as a percentage of a total of 26 identified FAs in whole blood. Analyses were quality-controlled according to DIN ISO 15189.

#### Echocardiography and other variables

In the Aldo-DHF Trial, clinical data were obtained and diagnostic procedures were done according to pre-defined standard operating procedures based on international guidelines [24]. Diastolic function on echocardiography was assessed in accordance with American Society of Echocardiography guidelines [27].

### Ethics

The Aldo-DHF Trial complies with the Declaration of Helsinki and principles of good clinical practice. All responsible ethics committees approved the study protocol. All participants gave written informed consent.

### Statistical analysis

Continuous variables are reported as mean ± standard deviation (SD) or median [interquartile range (IQR)], according to normality of distribution. Discrete variables are presented as absolute and relative frequencies. Bivariate correlations were assessed by Pearson’s correlation coefficient (r) to evaluate associations of the O3I and prespecified O3-FA (ALA, EPA, DHA) with cardiometabolic risk markers, echocardiographic markers of LVDF and neurohumoral activation. Partial correlations adjusting for heart failure medication (ACE inhibitors, AT1-antagonists, betablockers, thiazide-diuretics, loop-diuretics, aldosterone-antagonists, other diuretics, calcium-channel-blockers), systolic and diastolic blood pressure (BP), and HbA1c were estimated. Independent samples t-test was used to compare E/e’ of the groups with an O3I < 4% and an O3I > 8%. These indices represent clinically established cut-offs for deficiency and optimal O3 status respectively. Multiple linear regression analyses, using sex and age as covariates, were used to describe the association of the O3I and prespecified O3-FA (ALA/EPA/DHA) with cardiometabolic risk markers, echocardiographic markers of LVDF and neurohumoral activation at baseline and at 12-month follow-up. Model A depicts the effects of the O3I (EPA + DHA), ALA, sex and age on the outcomes, Model B shows the effects of ALA, EPA, DHA, sex and age on the outcomes. Model C (male subgroup) and D (female subgroup) depict sex-specific analyses within the subgroups of sex for all outcomes. A significance level of *α* = 5% was used for all tests. Considering our cohort of 404 patients, the probability (= power) of detecting an association between two statistical variables was 85% assuming a true correlation of *r* = 0.15. As all tests were hypothesis generating without confirmatory interpretation, no correction was applied to counteract the problem of multiple comparisons. All statistical analyses were performed using IBM SPSS Statistics for Windows, version 25 (IBM Corp., Armonk, NY, USA).

## Results

### Study population

**Fig. 1:**
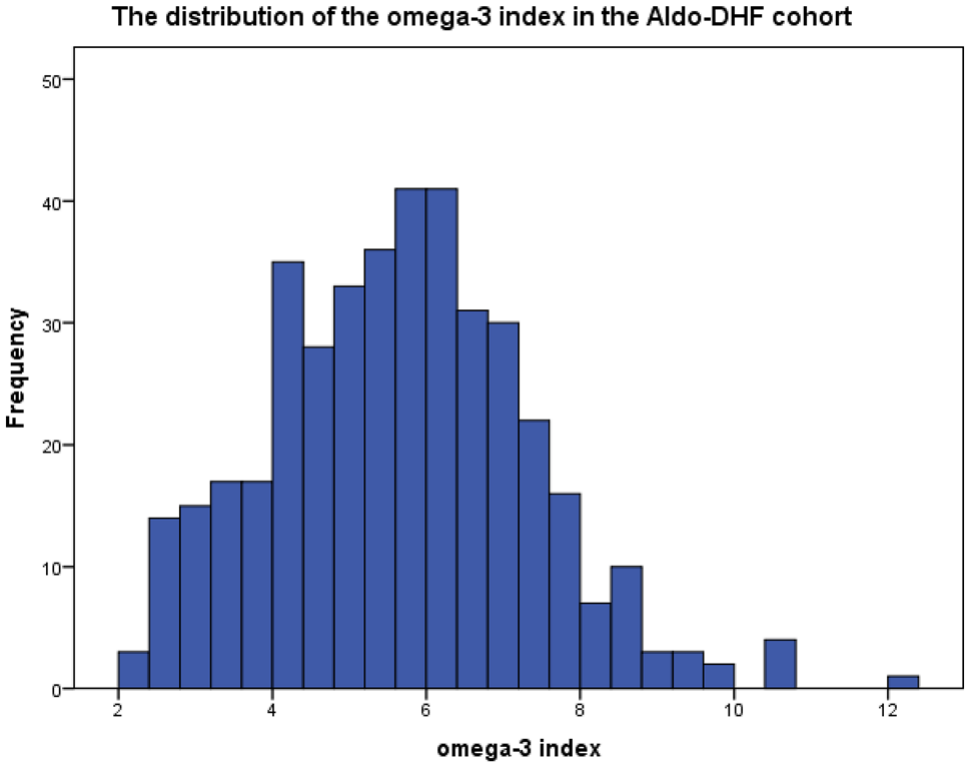
The distribution of the O3I in the Aldo-DHF Cohort. O3I: n 404, mean 5.65%, SD 1.676, min 2.19%, max 12.11%

Baseline characteristics are shown in Table 1. Mean O3I at baseline was 5.7 ± 1.7% (minimum 2.19%; maximum 12.11%) as depicted in Fig. 1. The O3I was below (< 8%), within (8–11%), or higher (> 11%) than the target range in in 374 (93%), 29 (7%) and in 1 (0.2%) patients respectively.

### O3I and LVEF

**Fig. 2:**
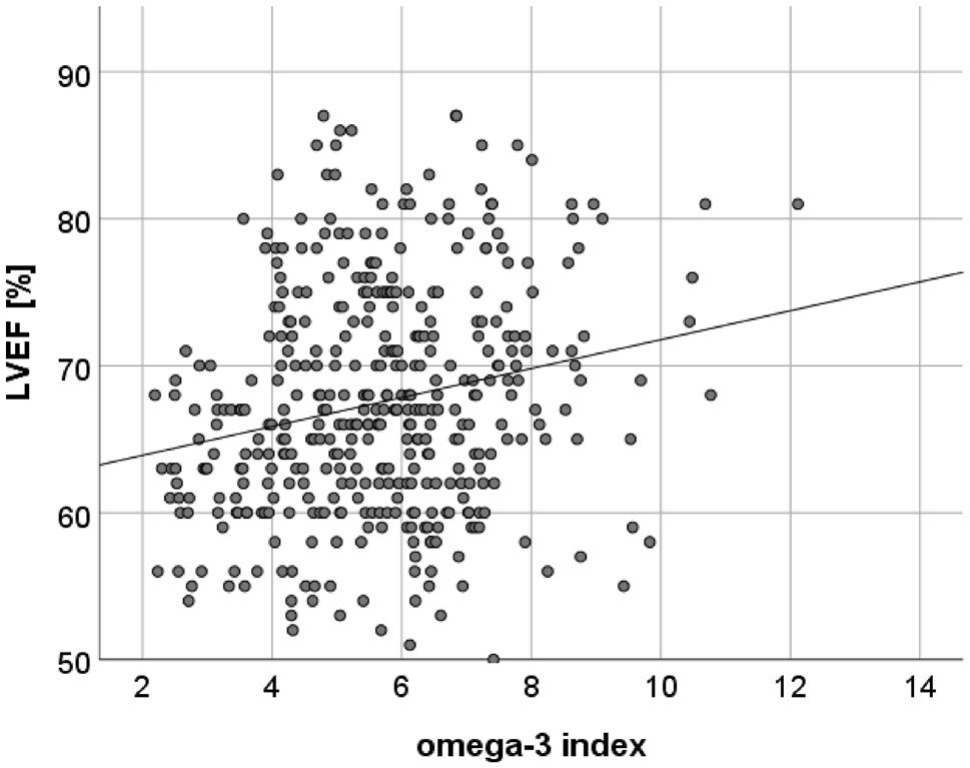
Scatter Plot showing correlations between the O3I and LVEF. The O3I showed a positive association with LVEF

O3I, EPA, and DHA showed a positive association with LVEF (*r* = 0.211, *p* < 0.001; *r* = 0.123, *p* = 0.013, and *r* = 0.226, *p* < 0.001, respectively) as shown in Fig. 2 and Supplementary Table 1. Furthermore, higher O3I at baseline was predictive of higher LVEF (*β* = 0.698, *p* = 0.007) at 12-month follow-up, with DHA mostly accounting for this association (Model B: *β* = 1.393, *p* = 0.003) as depicted in Supplementary Table 4.

### O3I and metabolic phenotype

**Fig. 3:**
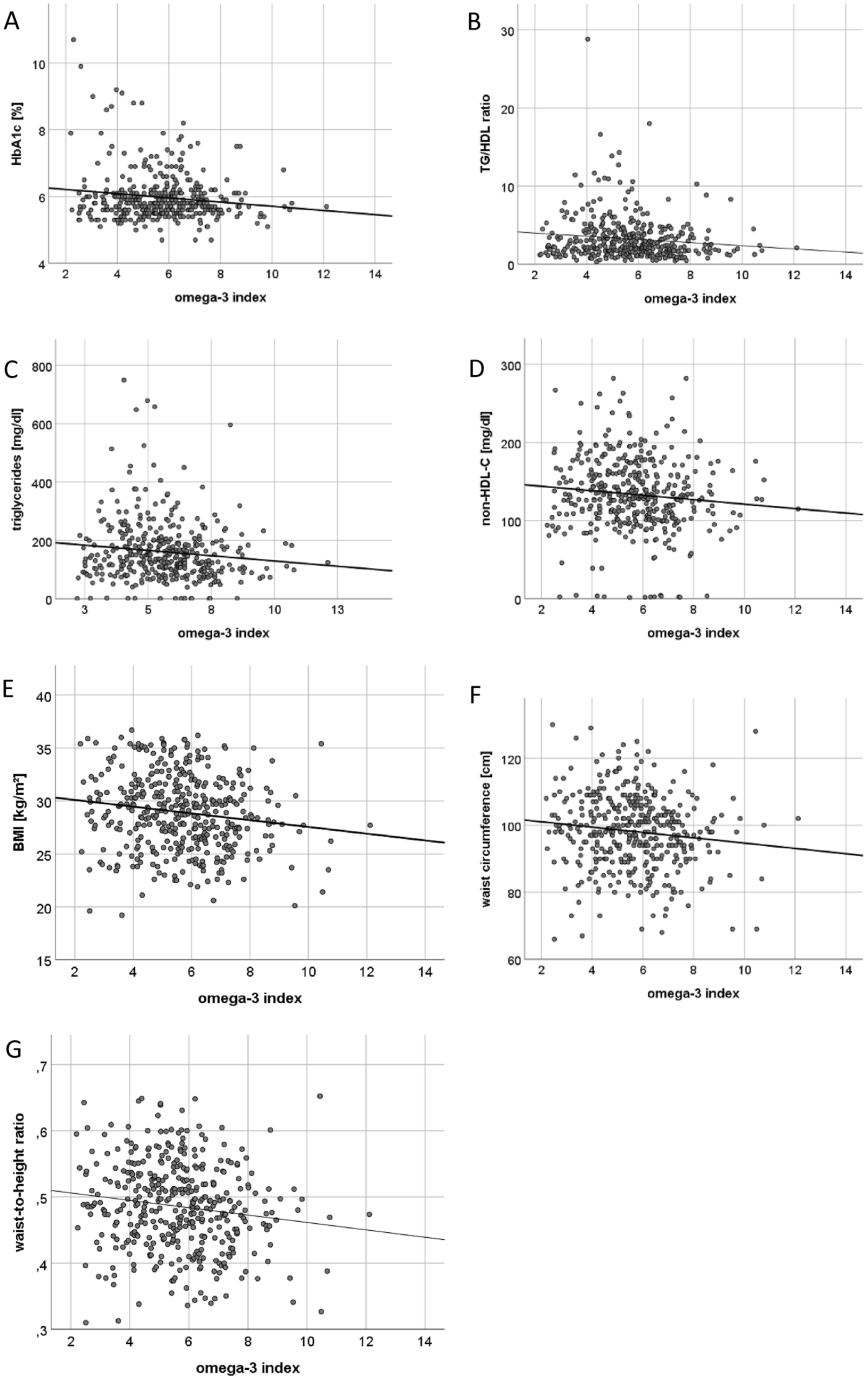
Scatter Plots showing correlations between the O3I and HbA1c (**A**), TAG/HDL-C ratio (**B**), Triglycerides (**C**), Non-HDL-C (**D**), Body Mass Index (**E**), WC (**F**) and Waist-to-height ratio (**G**). The Omega-3 index was inversely associated with HbA1c (**A**), TAG/HDL-C ratio (**B**), Triglycerides (**C**), Non-HDL-C (**D**), BMI (**E**), WC (**F**) and WHtR (**G**)

Higher O3I at baseline was broadly associated with a more favorable cardiometabolic risk profile at baseline as depicted in Multipanel Fig. 3A–G and Table 2. The O3I was inversely associated with HbA1c (*r* = − 0.139, *p* = 0.006), TG/HDL-C ratio (*r* = − 0.12, *p* = 0.017), triglycerides (*r* = − 0.117, *p* = 0.02), non-HDL-C (*r* = − 0.101, *p* = 0.044), BMI (*r* = − 0.149, *p* = 0.003), waist circumference (WC) (*r* = − 0.121, *p* = 0.015), waist-to-height ratio (WHtR) (*r* = − 0.141, *p* = 0.005) and GGT (*r* = − 0.125, *p* = 0.012).

**Table 2:** Associations between the O3I/individual O3-FA, metabolic phenotype, and BMI/anthropometric markers of truncal adiposity

		HbA1c	TG/HDL-C ratio	Triglycerides	Non-HDL-C	GGT	BMI	WC	WHtR
ALA C18:3n3	Pearson’s correlation coefficient	− 0.055	0.178^**^	0.179^**^	− 0.003	− 0.053	− 0.072	0.066	− 0.071
	*p*-value*	0.277	*p* < 0.001	*p* < 0.001	0.955	0.293	0.148	0.185	0.152
	*N*	392	397	399	398	402	404	403	404
EPA C20:5n3	Pearson’s correlation coefficient	− 0.079	− 0.124*	− 0.091	− 0.043	− 0.058	− 0.178**	− 0.117*	− 0.173**
	*p*-value*	0.121	0.014	0.070	0.395	0.246	*p* < 0.001	0.019	*p* < 0.001
	*N*	392	397	399	398	402	404	403	404
DHA C22:6n3	Pearson’s correlation coefficient	− 0.150**	− 0.107*	− 0.116*	− 0.115*	− 0.140**	− 0.124*	− 0.071	− 0.115*
	*p*-value*	0.003	0.033	0.020	0.022	0.005	0.013	0.153	0.021
	*N*	392	397	399	398	402	404	403	404
Omega-3 Index	Pearson’s correlation coefficient	− 0.139**	− 0.120*	− 0.117*	− 0.101*	− 0.125*	− 0.149**	− 0.090	− 0.141**
	*p*-value*	0.006	0.017	0.020	0.044	0.012	0.003	0.070	0.005
	*N*	392	397	399	398	402	404	403	404

		Systolic blood pressure	Diastolic blood pressure	Heart rate	HDL-C	LDL-C	ALT (GPT)	AST (GOT)
ALA C18:3n3	Pearson’s correlation coefficient	0.014	− 0.107*	0.011	− 0.106*	− 0.020	− 0.098*	− 0.042
	*p*-value*	0.776	0.032	0.819	0.034	0.685	0.050	0.402
	*N*	404	404	404	400	393	402	402
EPA C20:5n3	Pearson’s correlation coefficient	− 0.011	− 0.074	− 0.109*	0.059	0.005	− 0.079	− 0.039
	*p*-value*	0.821	0.140	0.028	0.242	0.919	0.115	0.439
	*N*	404	404	404	400	393	402	402
DHA C22:6n3	Pearson’s correlation coefficient	− 0.024	− 0.097	− 0.068	0.028	− 0.048	− 0.079	− 0.054
	*p*-value*	0.633	0.052	0.175	0.582	0.344	0.115	0.282
	*N*	404	404	404	400	393	402	402
Omega-3 Index	Pearson’s correlation coefficient	− 0.022	− 0.097	− 0.085	0.039	− 0.035	− 0.084	− 0.053
	*p*-value*	0.663	0.052	0.087	0.435	0.487	0.091	0.288
	*N*	404	404	404	400	393	402	402

The numbers of patients available for every variable is indicated

*All tests were performed 2-sided

After adjusting for sex and age results were very similar, further substantiating our findings. The inverse association of the O3I (Model A) persisted with HbA1c (*β* = − 0.060, *p* = 0.014), TG/HDL-C ratio (*β* = − 0.287, *p* = 0.001), triglycerides (*β* = − 10.187, *p* = 0.002), BMI (*β* = − 0.269, *p* = 0.016), WC (*β* = − 0.942, *p* = 0.002), and WHtR (*β* = − 0.004, *p* = 0.034) but associations with GGT (*β* = − 2.388, *p* = 0.054) and non-HDL-C (*β* = − 2.474, *p* = 0.096) were no longer significant. Analysis of individual FA (Model B) showed that DHA, but not EPA, was inversely correlated with HbA1c (*β* = − 0.114, *p* = 0.009) and non-HDL-C (*β* = − 5.681, *p* = 0.034) at baseline. EPA, but not DHA, was inversely correlated with TG/HDL-C ratio (*β* = − 1.177, *p* = 0.004), BMI (*β* = − 1.239, *p* = 0.018), WC (*β* = − 2.926, *p* = 0.039), and WHtR (*β* = − 0.024, *p* = 0.008) at baseline.

The O3I at baseline predicted a lower BMI (*β* = − 0.274, *p* = 0.026), WC (*β* = − 1.020, *p* = 0.002), and WHtR (*β* = − 0.006, *p* = 0.001) at 12-month follow-up. Sex was a significant predictor of HbA1c, TG/HDL-C ratio, triglycerides, non-HDL-C, WC, and WHtR but not for BMI.

Associations between ALA, EPA and DHA are depicted in Supplementary Table 4.

No association was found between the O3I and blood pressure (BP) under medication [(systolic (*r* = − 0.022, *p* = 0.663), diastolic (*r* = − 0.097, *p* = 0.052)], heart rate (HR) under medication (*r* = − 0.085, *p* = 0.087), HDL-C (*r* = 0.039, *p* = 0.435), LDL-C (*r* = − 0.035, *p* = 0.487), and transaminases [(ASAT (*r* = − 0.053, *p* = 0.288), ALAT (*r* = − 0.084, *p* = 0.591)]. Applying multiple linear regression analyses with covariates sex and age did not change these results at baseline nor were there significant associations at 12-month follow-up. Furthermore, the association between the O3I and GGT was no longer significant.

### O3I and functional capacity

**Fig. 4:**
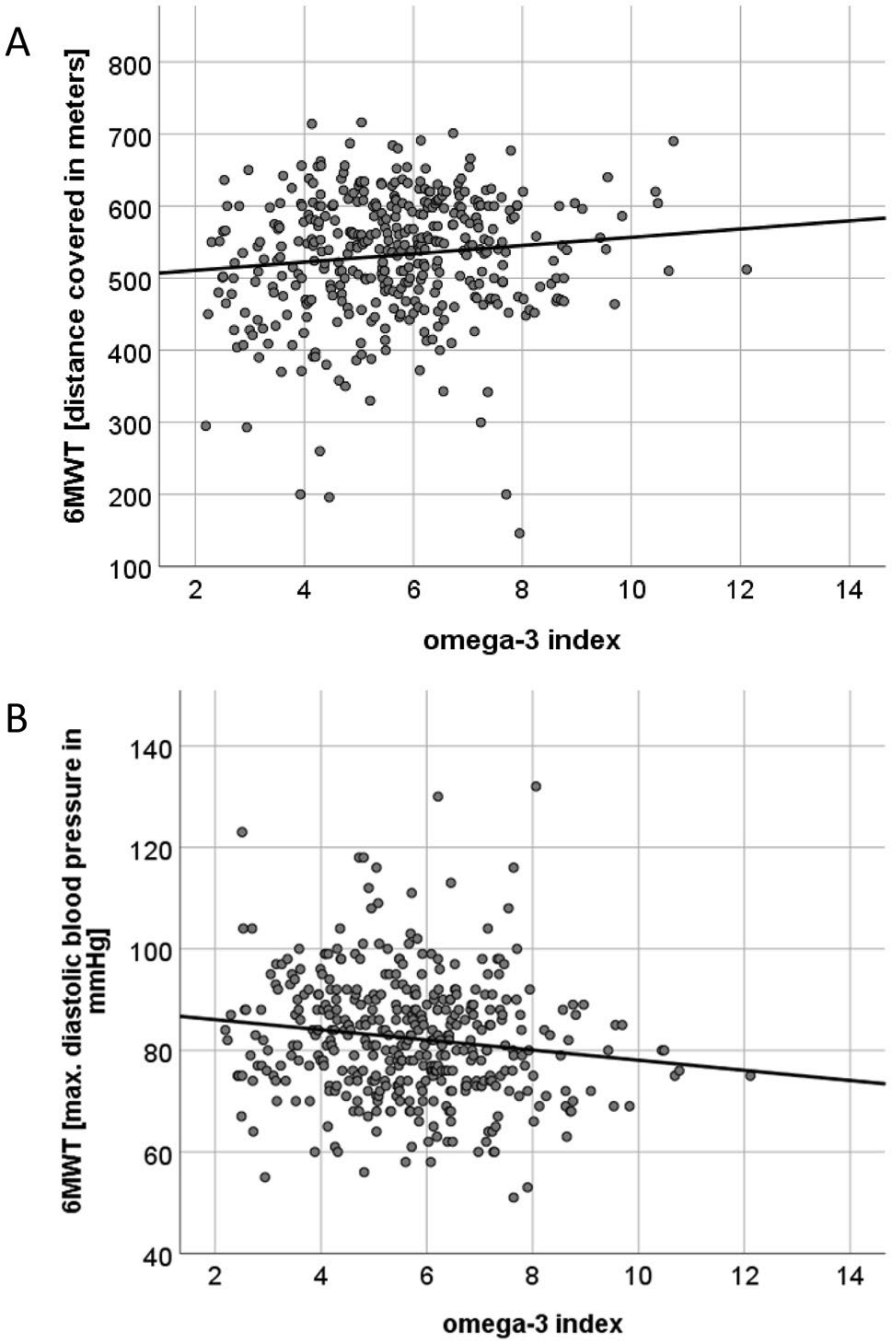
Scatter Plots showing correlations between the O3I and distance covered during the 6 MWT (**A**) and Maximal Diastolic BP during the 6MWT (**B**). The O3I showed a positive association with submaximal aerobic capacity (**A**) and was inversely associated with maximal diastolic BP during the 6MWT (**B**)

O3I was associated with a higher distance covered (*r* = 0.113, *p* = 0.023), and lower maximal diastolic BP during the 6-min walk test (6 MWT) (*r* = − 0.138, *p* = 0.006) as depicted in Multipanel Fig. 4A, B and Supplementary Table 2. Using sex and age as covariates, higher O3I at baseline predicted submaximal aerobic capacity (*β* = 15.614, *p* < 0,001), and maximal aerobic capacity (VO2peak) (*β* = 0.399, *p* = 0.005) at 12-month follow-up as shown in Supplementary Table 4.

### O3I and LVDF

The O3I showed no correlation with markers for left ventricular relaxation or other surrogates for LVDF: E/A ratio (*r* = − 0.031, *p* = 0.55), deceleration time (*r* = 0.019, *p* = 0.70), *E*/*e′* (*r* = − 0.029, *p* = 0.56), and left atrial volume index (LAVI) (*r* = − 0.029, *p* = 0.571). Using logarithmic transformation, the association of the O3I and neurohumoral activation (NT-proBNP) was not significant (*r* = 0.062, *p* = 0.221).

Using as covariates sex and age, the O3I at baseline was not significantly associated with E/A ratio, deceleration time, *E*/*e′*, and NT-proBNP at baseline or 12-month follow-up. The major statistically significant determinant for *E*/*e′* (*β* = − 0.048, *p* < 0.001 and *β* = − 0.046, *p* < 0.001) and NT-proBNP (*β* = 0.019, *p* < 0.001 and *β* = 0.055, *p* < 0.001) at baseline and follow-up, respectively, was age as shown in Supplementary Table 4.

Partial correlations adjusting for heart failure medication and HbA1c did not alter our results.

The comparison of *E*/*e′* in an O3 deficient versus O3 sufficient group [i.e., an O3I < 4% (*n* = 64) vs an O3I > 8% (*n* = 30); (*t*(92)) = 0.61, *p* = 0.54)] showed no statistically significant difference.

### Association between individual O3-FA and LVDF

Using Pearson’s *r*, the plant-derived alpha-linolenic acid (ALA; C18:3n3) was very weakly (*r* = 0.184, *p* < 0.001) and EPA, DHA, or the O3I (*r* = 0.096, *p* = 0.057 and *r* = 0.042, *p* = 0.407) showed no significant correlation with levels of NT-proBNP. We did not observe a significant association between ALA, EPA, and DHA and echocardiographic measures of LVDF. Adjusting for heart failure medication, systolic and diastolic blood pressure, and HbA1c did not alter our results. All correlations coefficients of the O3I and individual FAs (ALA, EPA, DHA) with LVDF and neurohumoral activation are depicted in Supplementary Table 3.

Using multiple linear regression analyses with covariates sex and age, ALA at baseline was associated with adverse effects on lipid phenotype at baseline TG/HDL-C ratio (Model A: *β* = 5.1, *p* < 0.001 and Model B: *β* = 5.809, *p* < 0.001), and triglycerides (Model A: *β* = 179.46, *p* < 0.001 and Model B: *β* = 191.793, *p* < 0.001). Higher levels of ALA predicted higher TG/HDL-C ratio (Model A: *β* = 3.613, *p* = 0.026 and Model B: *β* = 3.689, *p* = 0.03) at 12-month follow-up. Furthermore, ALA was associated with significantly higher NT-proBNP at baseline (Model A: *β* = 0.386, *p* = 0.022 and Model B: *β* = 0.355, *p* = 0.043), predicted higher NT-proBNP at 12 month follow-up (Model A: *β* = 1.034, *p* = 0.010 and Model B: *β* = 0.892, *p* = 0.033) and was associated with higher *E*/*e*′ (Model A: *β* = 1.521, *p* = 0.027 and Model B: *β* = 1.402, *p* = 0.026) at baseline.

### Sex-specific analyses

All models were adjusted using sex as a covariate and overall, sex had a significant influence in several models but without turning the results in a specific direction regarding the fatty acid. Furthermore, sex-specific analyses within the gender subgroups were done for all outcomes, showing sex-specific differences regarding the association of the O3I with:

1. Lipid phenotype: In men, but not in women, the O3I was significantly associated with a more favorable lipid phenotype [men: (TG/HDL-C ratio (*β* = − 0.458, *p* = 0.001), triglycerides (*β* = − 17.675, *p* < 0.001), non-HDL-C (*β* = − 6.138, *p* = 0.003) vs women: (TG/HDL-C ratio (*β* = − 0.155, *p* = 0.135), triglycerides (*β* = − 4.132, *p* = 0.299), non-HDL-C (*β* = 0.333, *p* = 0.872)]. Of note, while there was no significant association between the O3I and LDL-C in the entire population, sex-specific analyses demonstrated a significant inverse association in men, but not in women at baseline and at 12 months follow-up: [LDL-C at baseline in men (*β* = − 3.960, *p* = 0.031), LDL-C at 12 months in men (*β* = − 4.383, *p* = 0.020)].
2. HbA1c: In women, but not in men, the O3I was significantly inversely associated with HbA1c [women: HbA1c (*β* = − 0.054, *p* = 0.046) vs men: HbA1c (*β* = − 0.065, *p* = 0.1)].
3. BMI and markers indicative of truncal adiposity: In women, but not in men, the O3I was inversely associated with BMI, waist circumference (WC) and waist-to-height ratio (WHtR) [women: BMI (*β* = − 0.471, *p* = 0.004), WC (*β* = − 1.417, *p* = 0.001), and WHtR (*β* = − 0.008, *p* = 0.002) vs men BMI (*β* = − 0.135, *p* = 0.353), WC (*β* = − 0.313, *p* = 0.433), and WHtR (*β* = − 0.003, *p* = 0.339)].
4. *E*/*e*′: In men, but not in women, the O3I at baseline was predictive of a lower *E*/*e*′at 12 months [men: (*E*/*e*′ (*β* = − 0.157, *p* = 0.039) vs women: (*E*/*e*′ (*β* = − 0.063, *p* = 0.416)].

Model C and D in Supplementary Table 4 depict sex-specific analyses.

## Discussion

This analysis shows omega-3 status in HFpEF patients and associations with cardiometabolic risk factors, exercise capacity, echocardiographic markers of left ventricular diastolic function, and neurohumoral activation as depicted in the *Graphic Abstract*.

### LVEF, LV-mass, and the O3I

Epidemiological and experimental studies as well as post-hoc analyses of randomized controlled trials (RCT) suggest that O3-FA are beneficial in chronic HF. In the Cardiovascular Health Study, higher circulating individual and total O3-FA concentrations correlated with a lower incidence of congestive HF in 2735 adults without heart disease [28]. Similarly, two double-blind, placebo controlled, RCT showed that in 6975 and 4574 patients with HF, O3-FA (1 g/day), but not rosuvastatin (10 mg/day), significantly decreased the two coprimary end points (death, and death or admission to hospital for cardiovascular reasons) [29]. Another study provided a small benefit regarding supplementation with 1 g O3-FA in terms of mortality and hospital admissions in patients with HF [30]. In patients with acute myocardial infarction (MI), treatment with high-dose O3-FA was associated with a reduction of adverse left ventricular remodeling, noninfarct myocardial fibrosis (ST2), and serum biomarkers of systemic and vascular inflammation (MPO, Lp-PLA2) [7]. For these reasons, treatment with O3-FA has been endorsed for treatment of HFrEF with a Class IIa and Class IIb recommendation by the AHA [18] and ESC [2] respectively. In line, our data suggest a positive but weak correlation of LVEF with the O3I, EPA and DHA. Furthermore, higher O3I at baseline was predictive of higher LVEF at 12-month follow-up, with DHA mostly accounting for this association.

### Metabolic phenotype and the O3I

#### Lipid metabolism

Mechanistically, O3-FA interfere with hepatic lipid metabolism by downregulating genes involved in de-novo lipogenesis (DNL), and thus deplete the hepatic pool of triglycerides [16]. Accordingly, high intakes of EPA&DHA have been demonstrated to reduce the expression of atherogenic dyslipidaemia. For example, O3-FA (> 1 g for at least 3 months) modulate particle distribution, phenotype and lipidome by reducing ApoB46 and ApoC3 [16, 31] and triglycerides [32] in patients with MetS. In concordance with these findings, we observed an inverse correlation of triglycerides, TG/HDL-C ratio, and non-HDL-C with the O3I. A higher TG/HDL-C ratio is associated with higher levels of remnant lipoprotein particle cholesterol, non-HDL-C and LDL density, reflecting an atherogenic lipid phenotype [33], and a higher risk plaque phenotype (thin-cap fibroatheroma) in patients with coronary artery disease [34–36]. Triglycerides show a causal and dose dependent association with ASCVD risk [37]. Collectively, this provides confidence that our findings indicate that a higher O3I is associated with a lower risk lipoprotein pattern. As expected, no inverse association was found between the O3I and LDL-C, which aligns with prior RCTs, where therapeutic doses of O3-FA (3 g/day) were associated with increases in LDL-C in patients with MetS [32] or had no effect on LDL-C in patients with nonalcoholic fatty liver disease (NAFLD) [38]. Of note, in the subgroup of patients with MetS, non-HDL-C is superior to LDL-C as a risk indicator for atherosclerotic cardiovascular disease risk [39, 40]. With regard to LDL-C it is worth noting that while there was no significant association with the O3I in the entire study population, sex-specific analyses demonstrated a significant inverse association in men, but not in women at baseline and at 12 months follow-up, suggesting that further research on O3-FA and lipid metabolism should be directed to the subgroup of men. Regarding the TG/HDL-C ratio, triglycerides, and non-HDL-C there was a significant inverse association with the O3I in the entire study cohort and in men, but not in women, overall further suggesting that the subgroup of men drive the correlation between the O3I and lipid metabolism.

#### Glucose metabolism

While O3-FA in therapeutic doses are recommended for managing hypertriglyceridemia [37], data on their impact on glucose metabolism are inconclusive. The majority of observational studies show an inverse association between O3-FA and insulin resistance [41]. Most human intervention studies and meta-analyses of RCTs failed to demonstrate a benefit of O3-FA in individuals living with insulin resistance or T2D [41, 42]. The tight pathophysiological link between NAFLD, hypertriglyceridemia and insulin resistance/T2D might offer biological plausibility for our finding of an inverse association of the O3I with blood glucose. Of note, HbA1c was inversely associated with the O3I in women, but not in men.

#### Surrogate markers for NAFLD

EPA&DHA influence hepatic triglyceride pool and lipoprotein metabolism by the following mechanisms: first they promote hepatic fatty acid oxidation by interfering with the PPAR (peroxisome proliferator-activated receptors) system, especially PPARα and second, they downregulate the expression of the two lipogenic transcription factors SREBP-1C and ChREBP [43]. In preclinical models, EPA&DHA are associated with anti-inflammatory and antisteatotic effects [44]. In humans, a meta-analysis on the effect of O3-FA supplementation (2–5 g per day) in patients with NAFLD or nonalcoholic steatohepatitis (NASH) in part replicated these results. O3-FA lowered liver fat content, and gamma-glutamyltransferase (GGT) levels but had no effect on alanine aminotransferase (ALT) and aspartate aminotransferase (AST) in patients with NAFLD/NASH. [38] Replicating these results, we observed an inverse association of the O3I with GGT, but not with transaminases.

#### BMI and anthropometric markers of truncal adiposity

BMI is inconsistently associated with outcomes, particularly in heart failure (i.e., obesity parardox) [45, 46]. Contrarily, anthropometric markers indicative of visceral adiposity such as WC [47] and WHtR [48, 49] are more reliable indicators of insulin resistance, adipose tissue distribution, cardiometabolic risk and/or high risk atherosclerosis [40]. We observed an inverse correlation of BMI, WC and WHtR with the O3I, indicating that in HFpEF patients, a higher omega-3 tissue status is associated with a lower risk metabolic phenotype. Further substantiating this notion, the O3I at baseline was predictive of a lower BMI, WC, and WHtR at 12-month follow-up. Interestingly, if analyzed within the subgroups of sex, the O3I was significantly associated with BMI and markers indicative of truncal adiposity only in women, but not in men, suggesting that the association in the entire cohort is driven by women. It might thus be advisable to concentrate further research on the association between body weight/composition and the O3I on women.

### Blood pressure and heart rate

Epidemiological data in normotensive young, healthy individuals showed a significant and clinically relevant inverse association of the O3I with systolic and diastolic BP [19]. This is in line meta-analytic evidence of RCTs that showed that EPA&DHA containing supplements decreased systolic/diastolic BP and HR [13]. As reported in Table 1, the vast majority of our patients were on anti-hypertensive medication and betablockers, likely to preclude us from confirming the inverse correlation between BP/HR and the O3I [13, 19].

### Functional capacity and the O3I

The O3I was associated with a higher distance covered and with a lower maximal diastolic BP during the 6MWT, reflecting better submaximal aerobic capacity. Furthermore, using as covariates sex and age, higher O3I at baseline predicted submaximal aerobic capacity, and maximal aerobic capacity (VO2peak) at 12-month follow-up. Data on the effect of O3-FA supplementation on exercise performance with regard to strength and anaerobic capacity in athletes are conflicting [50] and no such data have been generated in HF patients to the best of our knowledge.

### Neurohumoral activation and O3I

We did not observe a significant association of NT-proBNP with the O3I or EPA&DHA. However, natriuretic peptides are used as an adjunct to identify severe, but not moderate or mild diastolic function [51]. This cohort depicts early stage HFpEF and the median NT-proBNP plasma level in Aldo-DHF (158 ng/L) was higher than in healthy age-matched controls [52] but lower than in previous analyses of HFpEF patients such as I-PRESERVE [53]. Using multiple linear regression analyses with covariates sex and age, higher blood levels of the plant-derived O3-FA ALA at baseline was associated with significantly higher NT-proBNP at baseline and predicted higher NT-proBNP at 12 month follow-up.

### Echocardiographic markers of LVDF and O3I

Membrane lipid environments determine cellular functions. Incorporation of EPA&DHA into membrane phospholipids has been reported to alter metabolic/physiochemical properties of cells/tissues such as fluidity/elasticity [16]. Tissue levels of O3-FA might thus impact on both early (energy-dependent) and late (compliance-dependent) left ventricular diastolic filling [16]. In line, a longitudinal study evaluating the effect of 4 g/day of ethyl ester concentrates of DHA vs placebo (4 g/day corn oil) on echocardiographic parameters of LVDF showed significantly improved left ventricular diastolic filling in the marine compared to the placebo group [54]. Another study evaluating the supplementation of 1 g omega-3 FA in 205 patients with HFrEF (EF < 40%) due to ischemic or dilated cardiomyopathy (NYHA I-III, EF < 40%) showed improved LVDF after 6 months of treatment compared to placebo. [6] Further to that, a double-blind, placebo controlled, cross-over trial evaluating the effect of an 8-week supplementation of 2 g O3-FA vs 2 g of olive oil in 31 patients with ischemic HF showed a significant improvement in LVDF on echocardiographic parameters in the marine group vs the placebo group (E/e’ ratio decreased by − 9.47% vs − 2.1%) [55]. Interestingly, using multiple linear regression analyses with covariates sex and age, higher blood levels of the plant-derived O3-FA ALA at baseline were associated with significantly higher NTproBNP and E/e’ at baseline and predicted higher NTproBNP at 12 month follow-up. We did not observe a significant relationship of the O3I (EPA + DHA) and echocardiographic and biochemical surrogates for left ventricular relaxation and left ventricular filling pressures. There are however, several limitations to consider that should provide caution against oversimplified inference when interpreting the association of O3I and LVDF in this cross-sectional analysis.

First, a striking difference is that prior studies have analyzed the change of echocardiographic parameters following supplementation of O3-FA longitudinally. It is thus tempting to speculate that sensitivity of echocardiographic markers might suffice for detection of changes in LVDF over time but are not appropriate for detection of inter-individual differences cross-sectionally. Consistent with this hypothesis, a prior cross-sectional analysis from the Aldo-DHF trial showed no significant association of exercise training with echocardiographic markers of diastolic function [51] whereas in the Ex-DHF pilot study, which analyzed the effect of exercise training on LVDF longitudinally, a positive effect of exercise training on diastolic function was reported [56]. Second, the spread of the O3I detected in the Aldo-DHF cohort might have been insufficient, a phenomenon that was observed earlier in another epidemiologic study [57, 58]. At baseline, the average O3I was 5.7 ± 1.7%, with values ranging from 2.19 to 12.11% as depicted in Fig. 1. Only 28 patients had an index > 8%. In the intervention trial that demonstrated a reduction in myocardial fibrosis with EPA&DHA, the O3I increased from 5.5 + 1.8% by 81%, i.e. to 10.0%. (13) If an O3I of 10% were needed to reduce myocardial fibrosis in HFrEF patients, the number of patients with such values in Aldo-DHF was probably too small to produce a positive result for this parameter, which might also have been true for the other parameters assessed in our study. An O3I-driven trial in patients with HFpEF similar to the one performed by Heydari et al. [7] would probably clarify this issue. Third, despite adjustment for potential confounders, we cannot exclude the possibility of residual confounding. Fourth, no data is available on O3-FA supplementation. Furthermore, in the light of the O3I at baseline being predictive of a lower *E*/*e*′at 12 months in men, but not in women or in the entire cohort, we suggest to focus on the subgroup of men regarding further research on the association of O3-FA and LVDF. Finally, the cross-sectional nature of this study design limits our ability to make a causal inference on the association of omega-3 status and LVDF. Collectively, these major limitations might justify an O3I-driven intervention trial that evaluates the effect of O3-FA supplementation on LVDF in patients with HFpEF longitudinally. *Graphic Abstract*.

Strengths of this analysis are the large sample size comprising 404 HFpEF patients who underwent detailed phenotypization. Furthermore, with a proportion of 53% of the patients included in Aldo-DHF being female, this analysis adequately reflects the gender distribution in HFpEF [4]. Third, O3-FA blood cell membrane levels reflect cardiac and other tissue EPA&DHA levels in the absence and presence of supplementation with EPA&DHA [20, 21].

## Conclusion

In patients with HFpEF, the O3I, which depicts proportions of the O3-FA EPA and DHA in erythrocyte phospholipids, was below the target range in 374 (93%) patients, indicating a deficit in EPA&DHA in this subgroup. Higher O3I was associated with a more favorable cardiometabolic risk profile, higher submaximal/maximal aerobic capacity and lower BMI/truncal adiposity but did not correlate with echocardiographic surrogate markers for left ventricular filling pressures, left ventricular relaxation or neurohumoral activation.

## Clinical perspectives

### Competency in patient care

Higher O3-FA tissue levels were associated with a more favorable cardiometabolic risk profile and with higher submaximal and maximal aerobic capacity in patients with HFpEF.

### Translational outlook

Prospective clinical studies are needed to test the hypothesis that the metabolic benefits derived from O3-FA translate into prognostic benefit in patients with HFpEF and to elucidate the mechanisms involved.

## Supplementary Information

Below is the link to the electronic supplementary material.

Supplementary file1 (DOCX 18 kb)

Supplementary file1 (PDF 666 kb)
