# Supplementary material for: Omega-3 fatty acid blood levels are inversely associated with cardiometabolic risk factors in HFpEF patients: the Aldo-DHF randomized controlled trial
Source: Clin Res Cardiol. 2021 Aug 28;111(3):308–21. doi: 10.1007/s00392-021-01925-9 (PMC8873063; doi:10.1007/s00392-021-01925-9)
Supplement: Supplementary file 1 — Supplementary file1 (DOCX 18 kb) [file 392_2021_1925_MOESM1_ESM.docx]

# **Supplements**

**Supplementary Table 1 Associations between the O3I/Individual O3-FA and LVEF / LV Mass. The numbers of patients available for every variable is indicated.**

|  | | LVEF | LV Mass |
| --- | --- | --- | --- |
| ALA C18:3n3 | Pearson's correlation coefficient | 0.007 | 0.021 |
|  | p-value* | 0.889 | 0.673 |
|  | N | 404 | 404 |
| EPA C20:5n3 | Pearson's correlation coefficient | 0.123^*^ | -0.007 |
|  | p-value* | 0.013 | 0.892 |
|  | N | 404 | 404 |
| DHA C22:6n3 | Pearson's correlation coefficient | 0.226^**^ | -0.023 |
|  | p-value* | p<0,001 | 0.647 |
|  | N | 404 | 404 |
| Omega-3 Index | Pearson's correlation coefficient | 0.211^**^ | -0.020 |
|  | p-value* | p<0,001 | 0.695 |
|  | N | 404 | 404 |

*All tests were performed 2-sided

**Supplementary Table 2 Associations between the O3I/Individual O3-FA and Functional Capacity. The numbers of patients available for every variable is indicated.**

|  | 6MWT distance covered (meters) | 6MWT systolic max. blood pressure | 6MWT diastolic max. blood pressure | VO2peak |
| --- | --- | --- | --- | --- |
| Pearson's correlation coefficient | -0.033 | 0.036 | -0.079 | -0.059 |
| p-value* | 0.508 | 0.473 | 0.113 | 0.234 |
| N | 403 | 401 | 401 | 404 |
| Pearson's correlation coefficient | 0.097 | 0.026 | -0.084 | 0.081 |
| p-value* | 0.052 | 0.602 | 0.095 | 0.103 |
| N | 403 | 401 | 401 | 404 |
| Pearson's correlation coefficient | 0.109^*^ | 0.005 | -0.147^**^ | -0.039 |
| p-value* | 0.029 | 0.926 | 0,003 | 0.438 |
| N | 403 | 401 | 401 | 404 |
| Pearson's correlation coefficient | 0.113^*^ | 0.012 | -0.138^**^ | -0.005 |
| p-value* | 0.023 | 0.817 | 0.006 | 0.919 |
| N | 403 | 401 | 401 | 404 |

*All tests were performed 2-sided

**Supplementary Table 3 Associations between the O3I/Individual O3-FA and echocardiographic parameters of LVDF/neurohumoral activation. The association of the O3I and NT-proBNP was calculated using logarithmic transformation (base-10 logarithm) for NT-proBNP. The numbers of patients available for every variable is indicated.**

|  | | E/A | deceleration time | E/e´ | LAVI | NT-proBNP |
| --- | --- | --- | --- | --- | --- | --- |
| ALA C18:3n3 | Pearson's correlation coefficient | 0.022 | -0.022 | 0.045 | -0.002 | 0.184^**^ |
|  | p-value* | 0.660 | 0.664 | 0.366 | 0.967 | p<0,001 |
|  | N | 387 | 404 | 403 | 391 | 392 |
| EPA C20:5n3 | Pearson's correlation coefficient | 0.011 | -0.033 | -0,051 | -0.027 | 0.096 |
|  | p-value* | 0.835 | 0.509 | 0.303 | 0.596 | 0.057 |
|  | N | 387 | 404 | 403 | 391 | 392 |
| DHA C22:6n3 | Pearson's correlation coefficient | -0.044 | 0.038 | -0,017 | -0.027 | 0.042 |
|  | p-value* | 0.389 | 0.448 | 0.727 | 0.598 | 0.407 |
|  | N | 387 | 404 | 403 | 391 | 392 |
| Omega-3 Index | Pearson's correlation coefficient | -0.031 | 0.019 | -0.029 | -0.029 | 0.062 |
|  | p-value* | 0.550 | 0.703 | 0.561 | 0.571 | 0.221 |
|  | N | 387 | 404 | 403 | 391 | 392 |

*All tests were performed 2-sided
