# Supplementary material for: Omega-3 fatty acid blood levels are inversely associated with cardiometabolic risk factors in HFpEF patients: the Aldo-DHF randomized controlled trial
Source: Clin Res Cardiol. 2021 Aug 28;111(3):308–21. doi: 10.1007/s00392-021-01925-9 (PMC8873063; doi:10.1007/s00392-021-01925-9)
Supplement: Supplementary file 2 — Supplementary file1 (PDF 666 kb) [file 392_2021_1925_MOESM2_ESM.pdf]

Table

Supplementary Table 4

Table A1: Outcome HbA1c

	Coefficient β	Std. Error	t-Value	p-Value
Omega-3 Index (%)	-0.060	0.024	-2.479	0.014
ALA	-0.107	0.300	-0.356	0.722
Sex	0.088	0.076	1.159	0.247
Age (years)	-0.001	0.005	-0.171	0.864

Table A2: Outcome TG/HDL-C ratio

	Coefficient β	Std. Error	t-Value	p-Value
Omega-3 Index (%)	-0.287	0.086	-3.324	0.001
ALA	5.100	1.114	4.577	<0.001
Sex	0.946	0.275	3.443	0.001
Age (years)	-0.001	0.019	-0.061	0.952

Table A3: Outcome Triglycerides (mg/dl)

	Coefficient β	Std. Error	t-Value	p-Value
Omega-3 Index (%)	-10.187	3.187	-3.196	0.002
ALA	179.460	41.032	4.374	<0.001
Sex	15.475	10.133	1.527	0.128
Age (years)	-0.059	0.701	-0.084	0.933

Table A4: Outcome Non-HDL-C (mg/dl)

	Coefficient β	Std. Error	t-Value	p-Value
Omega-3 Index (%)	-2.474	1.481	-1.671	0.096
ALA	5.119	18.780	0.273	0.785
Sex	-13.794	4.708	-2.930	0.004
Age (years)	-0.364	0.326	-1.116	0.265

Table A5: Outcome Body mass index

	Coefficient β	Std. Error	t-Value	p-Value
Omega-3 Index (%)	-0.269	0.112	-2.410	0.016

ALA	-0.738	1.424	-0.518	0.605
Sex	0.250	0.356	0.703	0.483
Age (years)	-0.028	0.025	-1.153	0.250

Table A6: Outcome Waist Circumference, (cm)

	Coefficient β	Std. Error	t-Value	p-Value
Omega-3 Index (%)	-0.942	0.301	-3.131	0.002
ALA	2.701	3.839	0.704	0.482
Sex	10.673	0.959	11.128	<0.001
Age (years)	0.076	0.067	1.143	0.254

Table A7: Outcome Waist-to-height ratio

	Coefficient β	Std. Error	t-Value	p-Value
Omega-3 Index (%)	-0.004	0.002	-2.131	0.034
ALA	0.010	0.025	0.393	0.694
Sex	0.045	0.006	7.269	<0.001
Age (years)	-0.001	0.000	-2.928	0.004

Table A8: Outcome E/e'

	Coefficient β	Std. Error	t-Value	p-Value
Omega-3 Index (%)	0.016	0.048	0.342	0.732
ALA	1.144	0.605	1.890	0.060
Sex	0.021	0.151	0.139	0.890
Age (years)	-0.048	0.011	-4.556	<0.001

Table A9: Outcome E/A velocity ratio

	Coefficient β	Std. Error	t-Value	p-Value
Omega-3 Index (%)	-0.003	0.011	-0.247	0.805
ALA	0.114	0.137	0.837	0.403
Sex	-0.006	0.034	-0.191	0.849

Age (years)	-0.004	0.002	-1.804	0.072

Table A10: Outcome Deceleration time, ms

	Coefficient β	Std. Error	t-Value	p-Value
Omega-3 Index (%)	0.393	1.967	0.200	0.842
ALA	-13.277	25.067	-0.530	0.597
Sex	9.927	6.266	1.584	0.114
Age (years)	0.416	0.435	0.956	0.340

Table A11: Outcome NT-proBNP, ng/L

	Coefficient β	Std. Error	t-Value	p-Value
Omega-3 Index (%)	0.003	0.013	0.256	0.798
ALA	0.386	0.168	2.298	0.022
Sex	-0.033	0.042	-0.778	0.437
Age (years)	0.019	0.003	6.551	<0.001

Table A12: Outcome Follow-Up HbA1c

	Coefficient β	Std. Error	t-Value	p-Value
Omega-3 Index (%)	-0.020	0.031	-0.641	0.522
ALA	0.392	0.386	1.016	0.310
Sex	0.228	0.096	2.365	0.019
Age (years)	-0.002	0.007	-0.313	0.754

Table A13: Outcome Follow-Up TG/HDL-C ratio

	Coefficient β	Std. Error	t-Value	p-Value
Omega-3 Index (%)	-0.135	0.129	-1.046	0.296
ALA	3.613	1.622	2.228	0.026
Sex	1.482	0.405	3.658	<0.001
Age (years)	-0.037	0.028	-1.310	0.191

Table A14: Outcome Follow-Up Triglycerides (mg/dl)

	Coefficient β	Std. Error	t-Value	p-Value

Omega-3 Index (%)	-2.459	3.802	-0.647	0.518
ALA	82.211	47.825	1.719	0.086
Sex	29.402	11.951	2.460	0.014
Age (years)	-0.940	0.826	-1.139	0.255

Table A15: Outcome Follow-Up Non-HDL-C (mg/dl)

	Coefficient β	Std. Error	t-Value	p-Value
Omega-3 Index (%)	-1.416	1.536	-0.922	0.357
ALA	-19.073	19.335	-0.986	0.325
Sex	-11.282	4.831	-2.335	0.020
Age (years)	-0.447	0.334	-1.338	0.182

Table A16: Outcome Follow-Up Body mass index

	Coefficient β	Std. Error	t-Value	p-Value
Omega-3 Index (%)	-0.274	0.122	-2.243	0.026
ALA	-0.350	1.543	-0.227	0.821
Sex	0.218	0.385	0.564	0.573
Age (years)	-0.043	0.027	-1.616	0.107

Table A17: Outcome Follow-Up Waist Circumference, (cm)

	Coefficient β	Std. Error	t-Value	p-Value
Omega-3 Index (%)	-1.020	0.329	-3.103	0.002
ALA	0.560	4.105	0.136	0.892
Sex	11.372	1.027	11.075	<0.001
Age (years)	-0.023	0.071	-0.321	0.748

Table A18: Outcome Follow-Up Waist-to-height ratio

	Coefficient β	Std. Error	t-Value	p-Value
Omega-3 Index (%)	-0.006	0.002	-3.246	0.001
ALA	-0.023	0.025	-0.922	0.357

Sex	0.018	0.006	2.902	0.004
Age (years)	0.001	0.000	1.814	0.070

Table A19: Outcome Follow-Up E/e'

	Coefficient β	Std. Error	t-Value	p-Value
Omega-3 Index (%)	-0.063	0.054	-1.160	0.247
ALA	1.521	0.684	2.223	0.027
Sex	0.198	0.171	1.158	0.248
Age (years)	-0.046	0.012	-3.869	<0.001

Table A20: Outcome Follow-Up E/A velocity ratio

	Coefficient β	Std. Error	t-Value	p-Value
Omega-3 Index (%)	-0.001	0.011	-0.136	0.892
ALA	0.127	0.139	0.912	0.362
Sex	-0.008	0.035	-0.225	0.822
Age (years)	-0.007	0.002	-2.883	0.004

Table A21: Outcome Follow-Up Deceleration time, ms

	Coefficient β	Std. Error	t-Value	p-Value
Omega-3 Index (%)	4.053	2.074	1.954	0.051
ALA	-3.834	26.189	-0.146	0.884
Sex	9.719	6.537	1.487	0.138
Age (years)	1.147	0.453	2.534	0.012

Table A22: Outcome Follow-Up NT-proBNP, ng/L

	Coefficient β	Std. Error	t-Value	p-Value
Omega-3 Index (%)	-0.040	0.033	-1.241	0.215
ALA	1.034	0.398	2.596	0.010
Sex	-0.053	0.101	-0.528	0.598
Age (years)	0.055	0.007	7.810	<0.001

Table A23: Outcome Systolic blood pressure, mm Hg

	Coefficient β	Std. Error	t-Value	p-Value
Omega-3 Index (%)	-0.363	0.570	-0.637	0.524
ALA	4.245	7.260	0.585	0.559
Sex	4.591	1.815	2.530	0.012
Age (years)	0.041	0.126	0.327	0.744

Table A24: Outcome Diastolic blood pressure, mm Hg

	Coefficient β	Std. Error	t-Value	p-Value
Omega-3 Index (%)	-0.052	0.327	-0.160	0.873
ALA	-2.376	4.163	-0.571	0.569
Sex	2.224	1.041	2.138	0.033
Age (years)	-0.411	0.072	-5.695	<0.001

Table A25: Outcome Heart rate, min

	Coefficient β	Std. Error	t-Value	p-Value
Omega-3 Index (%)	0.192	0.356	0.540	0.589
ALA	3.472	4.537	0.765	0.445
Sex	-1.973	1.134	-1.739	0.083
Age (years)	-0.209	0.079	-2.650	0.008

Table A26: Outcome 6 MWT, (Distance covered) meters

	Coefficient β	Std. Error	t-Value	p-Value
Omega-3 Index (%)	11.093	2.396	4.629	<0.001
ALA	21.369	31.045	0.688	0.492
Sex	38.525	7.638	5.044	<0.001
Age (years)	-4.413	0.530	-8.323	<0.001

Table A27: Outcome LDL-C (mg/dl)

	Coefficient β	Std. Error	t-Value	p-Value

Omega-3 Index (%)	-0.260	1.331	-0.195	0.845
ALA	-5.451	16.859	-0.323	0.747
Sex	-12.092	4.226	-2.861	0.004
Age (years)	-0.409	0.293	-1.396	0.163

Table A28: Outcome ASAT

	Coefficient β	Std. Error	t-Value	p-Value
Omega-3 Index (%)	-0.118	0.299	-0.393	0.694
ALA	0.164	3.803	0.043	0.966
Sex	2.441	0.953	2.562	0.011
Age (years)	-0.145	0.066	-2.204	0.028

Table A29: Outcome ALAT

	Coefficient β	Std. Error	t-Value	p-Value
Omega-3 Index (%)	-0.098	0.450	-0.218	0.828
ALA	-3.039	5.721	-0.531	0.596
Sex	4.154	1.433	2.898	0.004
Age (years)	-0.458	0.099	-4.616	<0.001

Table A30: Outcome GGT

	Coefficient β	Std. Error	t-Value	p-Value
Omega-3 Index (%)	-2.388	1.238	-1.930	0.054
ALA	0.691	15.730	0.044	0.965
Sex	9.590	3.941	2.433	0.015
Age (years)	-0.465	0.273	-1.702	0.089

Table A31: Outcome LV ejection fraction, %

	Coefficient β	Std. Error	t-Value	p-Value
Omega-3 Index (%)	0.987	0.239	4.136	<0.001
ALA	-3.849	3.043	-1.265	0.207
Sex	-2.091	0.761	-2.749	0.006
Age (years)	0.048	0.053	0.911	0.363

Table A32: Outcome VO2peak

	Coefficient β	Std. Error	t-Value	p-Value
Omega-3 Index (%)	0.114	0.099	1.153	0.250
ALA	0.349	1.263	0.277	0.782
Sex	2.218	0.316	7.027	<0.001
Age (years)	-0.101	0.022	-4.587	<0.001

Table A33: Outcome Follow-Up Systolic blood pressure, mm Hg

	Coefficient β	Std. Error	t-Value	p-Value
Omega-3 Index (%)	-0.284	0.552	-0.516	0.606
ALA	2.873	6.927	0.415	0.679
Sex	4.427	1.731	2.558	0.011
Age (years)	-0.140	0.120	-1.166	0.244

Table A34: Outcome Follow-Up Diastolic blood pressure, mm Hg

	Coefficient β	Std. Error	t-Value	p-Value
Omega-3 Index (%)	-0.341	0.330	-1.033	0.302
ALA	-3.830	4.138	-0.926	0.355
Sex	0.634	1.034	0.613	0.540
Age (years)	-0.413	0.072	-5.765	<0.001

Table A35: Outcome Follow-Up Heart rate, min

	Coefficient β	Std. Error	t-Value	p-Value
Omega-3 Index (%)	-0.495	0.365	-1.356	0.176
ALA	-1.565	4.579	-0.342	0.733
Sex	0.067	1.144	0.058	0.954
Age (years)	-0.052	0.079	-0.659	0.510

Table A36: Outcome Follow-Up 6 MWT, (Distance covered) meters

	Coefficient β	Std. Error	t-Value	p-Value

Omega-3 Index (%)	15.614	2.913	5.360	<0.001
ALA	19.483	36.117	0.539	0.590
Sex	33.617	9.162	3.669	<0.001
Age (years)	-5.204	0.639	-8.149	<0.001

Table A37: Outcome Follow-Up LDL-C (mg/dl)

	Coefficient β	Std. Error	t-Value	p-Value
Omega-3 Index (%)	0.116	1.366	0.085	0.933
ALA	-25.786	17.145	-1.504	0.133
Sex	-12.819	4.295	-2.985	0.003
Age (years)	-0.283	0.296	-0.954	0.341

Table A38: Outcome Follow-Up ASAT

	Coefficient β	Std. Error	t-Value	p-Value
Omega-3 Index (%)	0.042	0.301	0.139	0.889
ALA	3.110	3.763	0.826	0.409
Sex	2.731	0.942	2.900	0.004
Age (years)	-0.159	0.065	-2.438	0.015

Table A39: Outcome Follow-Up ALAT

	Coefficient β	Std. Error	t-Value	p-Value
Omega-3 Index (%)	-0.061	0.432	-0.141	0.888
ALA	2.238	5.408	0.414	0.679
Sex	3.883	1.350	2.875	0.004
Age (years)	-0.531	0.094	-5.677	<0.001

Table A40: Outcome Follow-Up GGT

	Coefficient β	Std. Error	t-Value	p-Value
Omega-3 Index (%)	-0.304	1.231	-0.247	0.805
ALA	8.413	15.371	0.547	0.584
Sex	7.842	3.851	2.036	0.042
Age (years)	-0.558	0.267	-2.090	0.037

Table A41: Outcome Follow-Up LV ejection fraction, %

	Coefficient β	Std. Error	t-Value	p-Value
Omega-3 Index (%)	0.698	0.259	2.696	0.007
ALA	-0.826	3.268	-0.253	0.801
Sex	-1.961	0.816	-2.404	0.017
Age (years)	0.038	0.056	0.678	0.498

Table A42: Outcome Follow-Up VO2peak

	Coefficient β	Std. Error	t-Value	p-Value
Omega-3 Index (%)	0.399	0.140	2.846	0.005
ALA	1.822	1.783	1.022	0.307
Sex	2.556	0.443	5.772	<0.001
Age (years)	-0.141	0.031	-4.570	<0.001

Table B1: Outcome HbA1c

	Coefficient β	Std. Error	t-Value	p-Value
ALA	-0.231	0.312	-0.739	0.460
EPA	0.091	0.111	0.816	0.415
DHA	-0.114	0.044	-2.615	0.009
Sex	0.086	0.076	1.134	0.258
Age (years)	-0.001	0.005	-0.101	0.920

Table B2: Outcome TG/HDL-C ratio

	Coefficient β	Std. Error	t-Value	p-Value
ALA	5.809	1.154	5.036	<0.001
EPA	-1.177	0.403	-2.922	0.004
DHA	-0.022	0.156	-0.142	0.887
Sex	0.954	0.273	3.488	0.001
Age (years)	-0.003	0.019	-0.159	0.874

Table B3: Outcome Triglycerides (mg/dl)

	Coefficient β	Std. Error	t-Value	p-Value
ALA	191.793	42.708	4.491	<0.001
EPA	-25.918	14.949	-1.734	0.084
DHA	-5.892	5.781	-1.019	0.309
Sex	15.624	10.133	1.542	0.124
Age (years)	-0.090	0.701	-0.128	0.898

Table B4: Outcome Non-HDL-C (mg/dl)

	Coefficient β	Std. Error	t-Value	p-Value
ALA	-2.346	19.489	-0.120	0.904
EPA	6.873	6.908	0.995	0.320
DHA	-5.681	2.677	-2.122	0.034
Sex	-13.916	4.703	-2.959	0.003
Age (years)	-0.345	0.326	-1.056	0.291

Table B5: Outcome Body mass index

	Coefficient β	Std. Error	t-Value	p-Value
ALA	0.008	1.475	0.005	0.996
EPA	-1.239	0.523	-2.369	0.018
DHA	0.021	0.201	0.103	0.918
Sex	0.260	0.355	0.734	0.463
Age (years)	-0.030	0.025	-1.229	0.220

Table B6: Outcome Waist Circumference, (cm)

	Coefficient β	Std. Error	t-Value	p-Value
ALA	4.218	3.984	1.059	0.290
EPA	-2.926	1.412	-2.072	0.039
DHA	-0.378	0.543	-0.697	0.486
Sex	10.692	0.958	11.160	<0.001
Age (years)	0.072	0.067	1.089	0.277

Table B7: Outcome Waist-to-height ratio

	Coefficient β	Std. Error	t-Value	p-Value
ALA	0.025	0.025	0.988	0.324
EPA	-0.024	0.009	-2.673	0.008
DHA	0.002	0.003	0.569	0.570
Sex	0.045	0.006	7.340	<0.001
Age (years)	-0.001	0.000	-3.028	0.003

Table B8: Outcome E/e'

	Coefficient β	Std. Error	t-Value	p-Value
ALA	1.402	0.628	2.233	0.026
EPA	-0.312	0.223	-1.402	0.162
DHA	0.123	0.086	1.431	0.153
Sex	0.025	0.151	0.162	0.871
Age (years)	-0.048	0.011	-4.619	<0.001

Table B9: Outcome E/A velocity ratio

	Coefficient β	Std. Error	t-Value	p-Value
ALA	0.074	0.142	0.519	0.604
EPA	0.049	0.050	0.988	0.324
DHA	-0.019	0.019	-1.013	0.312
Sex	-0.007	0.034	-0.215	0.830
Age (years)	-0.004	0.002	-1.763	0.079

Table B10: Outcome Deceleration time, ms

	Coefficient β	Std. Error	t-Value	p-Value
ALA	-1.764	25.981	-0.068	0.946
EPA	-14.313	9.218	-1.553	0.121
DHA	5.132	3.547	1.447	0.149
Sex	10.085	6.253	1.613	0.108
Age (years)	0.388	0.434	0.893	0.372

Table B11: Outcome NT-proBNP, ng/L

	Coefficient β	Std. Error	t-Value	p-Value
ALA	0.355	0.174	2.032	0.043
EPA	0.043	0.062	0.699	0.485
DHA	-0.009	0.024	-0.384	0.701
Sex	-0.033	0.042	-0.791	0.430
Age (years)	0.019	0.003	6.568	<0.001

Table B12: Outcome Follow-Up HbA1c

	Coefficient β	Std. Error	t-Value	p-Value
ALA	0.404	0.404	1.001	0.318
EPA	-0.035	0.143	-0.248	0.804
DHA	-0.016	0.055	-0.292	0.771
Sex	0.228	0.097	2.364	0.019
Age (years)	-0.002	0.007	-0.318	0.751

Table B13: Outcome Follow-Up TG/HDL-C ratio

	Coefficient β	Std. Error	t-Value	p-Value
ALA	3.689	1.696	2.175	0.030
EPA	-0.233	0.602	-0.388	0.699
DHA	-0.113	0.233	-0.486	0.628
Sex	1.484	0.406	3.656	<0.001
Age (years)	-0.037	0.028	-1.315	0.189

Table B14: Outcome Follow-Up Triglycerides (mg/dl)

	Coefficient β	Std. Error	t-Value	p-Value
ALA	79.294	50.076	1.583	0.114
EPA	0.843	17.762	0.047	0.962
DHA	-3.710	6.872	-0.540	0.590
Sex	29.316	11.974	2.448	0.015
Age (years)	-0.933	0.828	-1.127	0.260

Table B15: Outcome Follow-Up Non-HDL-C (mg/dl)

	Coefficient β	Std. Error	t-Value	p-Value
ALA	-19.422	20.252	-0.959	0.338
EPA	-1.088	7.170	-0.152	0.879
DHA	-1.630	2.771	-0.588	0.557
Sex	-11.293	4.841	-2.333	0.020
Age (years)	-0.445	0.335	-1.331	0.184

Table B16: Outcome Follow-Up Body mass index

	Coefficient β	Std. Error	t-Value	p-Value
ALA	0.131	1.616	0.081	0.935
EPA	-0.850	0.573	-1.483	0.139
DHA	-0.111	0.221	-0.505	0.614
Sex	0.234	0.386	0.605	0.545
Age (years)	-0.045	0.027	-1.664	0.097

Table B17: Outcome Follow-Up Waist Circumference, (cm)

	Coefficient β	Std. Error	t-Value	p-Value
ALA	1.337	4.307	0.310	0.756
EPA	-1.973	1.526	-1.293	0.197
DHA	-0.789	0.593	-1.332	0.184
Sex	11.401	1.029	11.082	<0.001
Age (years)	-0.025	0.071	-0.353	0.725

Table B18: Outcome Follow-Up Waist-to-height ratio

	Coefficient β	Std. Error	t-Value	p-Value
ALA	-0.023	0.026	-0.881	0.379
EPA	-0.007	0.009	-0.731	0.465
DHA	-0.007	0.004	-1.909	0.057
Sex	0.018	0.006	2.894	0.004
Age (years)	0.001	0.000	1.810	0.071

Table B19: Outcome Follow-Up E/e'

	Coefficient β	Std. Error	t-Value	p-Value
ALA	1.379	0.717	1.924	0.055
EPA	0.099	0.254	0.389	0.698
DHA	-0.119	0.098	-1.221	0.223
Sex	0.193	0.171	1.128	0.260
Age (years)	-0.045	0.012	-3.828	<0.001

Table B20: Outcome Follow-Up E/A velocity ratio

	Coefficient β	Std. Error	t-Value	p-Value
ALA	0.117	0.146	0.803	0.423
EPA	0.010	0.052	0.196	0.844
DHA	-0.005	0.020	-0.268	0.789
Sex	-0.008	0.035	-0.234	0.815
Age (years)	-0.007	0.002	-2.859	0.004

Table B21: Outcome Follow-Up Deceleration time, ms

	Coefficient β	Std. Error	t-Value	p-Value
ALA	-0.079	27.448	-0.003	0.998
EPA	-0.087	9.720	-0.009	0.993
DHA	5.686	3.742	1.520	0.129
Sex	9.845	6.550	1.503	0.134
Age (years)	1.136	0.454	2.505	0.013

Table B22: Outcome Follow-Up NT-proBNP, ng/L

	Coefficient β	Std. Error	t-Value	p-Value
ALA	0.892	0.416	2.143	0.033
EPA	0.129	0.150	0.859	0.391
DHA	-0.098	0.058	-1.680	0.094
Sex	-0.058	0.101	-0.575	0.566
Age (years)	0.056	0.007	7.864	<0.001

Table B23: Outcome Systolic blood pressure, mm Hg

	Coefficient β	Std. Error	t-Value	p-Value
ALA	4.019	7.550	0.532	0.595
EPA	-0.095	2.679	-0.035	0.972
DHA	-0.476	1.031	-0.462	0.644
Sex	4.587	1.817	2.524	0.012
Age (years)	0.042	0.126	0.330	0.741

Table B24: Outcome Diastolic blood pressure, mm Hg

	Coefficient β	Std. Error	t-Value	p-Value
ALA	-2.955	4.328	-0.683	0.495
EPA	0.686	1.535	0.447	0.655
DHA	-0.293	0.591	-0.496	0.620
Sex	2.216	1.042	2.128	0.034
Age (years)	-0.410	0.072	-5.666	<0.001

Table B25: Outcome Heart rate, min

	Coefficient β	Std. Error	t-Value	p-Value
ALA	5.702	4.700	1.213	0.226
EPA	-2.650	1.667	-1.589	0.113
DHA	1.117	0.642	1.741	0.082
Sex	-1.942	1.131	-1.717	0.087
Age (years)	-0.214	0.079	-2.724	0.007

Table B26: Outcome 6 MWT, (Distance covered) meters

	Coefficient β	Std. Error	t-Value	p-Value
ALA	20.320	32.333	0.628	0.530
EPA	13.030	11.287	1.154	0.249
DHA	11.320	4.339	2.609	0.009
Sex	38.512	7.648	5.035	<0.001
Age (years)	-4.410	0.531	-8.301	<0.001

Table B27: Outcome LDL-C (mg/dl)

	Coefficient β	Std. Error	t-Value	p-Value
ALA	-11.633	17.505	-0.665	0.507
EPA	7.541	6.185	1.219	0.224
DHA	-2.799	2.401	-1.166	0.244
Sex	-12.157	4.222	-2.879	0.004
Age (years)	-0.392	0.293	-1.339	0.181

Table B28: Outcome ASAT

	Coefficient β	Std. Error	t-Value	p-Value
ALA	-0.080	3.957	-0.020	0.984
EPA	0.186	1.407	0.132	0.895
DHA	-0.224	0.543	-0.413	0.680
Sex	2.437	0.954	2.554	0.011
Age (years)	-0.145	0.066	-2.190	0.029

Table B29: Outcome ALAT

	Coefficient β	Std. Error	t-Value	p-Value
ALA	-2.839	5.953	-0.477	0.634
EPA	-0.359	2.117	-0.169	0.866
DHA	-0.021	0.817	-0.026	0.979
Sex	4.157	1.436	2.896	0.004
Age (years)	-0.459	0.100	-4.612	<0.001

Table B30: Outcome GGT

	Coefficient β	Std. Error	t-Value	p-Value
ALA	-7.055	16.307	-0.433	0.665
EPA	7.329	5.800	1.264	0.207
DHA	-5.697	2.238	-2.545	0.011
Sex	9.456	3.932	2.405	0.017
Age (years)	-0.445	0.273	-1.633	0.103

Table B31: Outcome LV ejection fraction, %

	Coefficient β	Std. Error	t-Value	p-Value
ALA	-2.461	3.154	-0.780	0.436
EPA	-0.731	1.119	-0.653	0.514
DHA	1.613	0.431	3.747	<0.001
Sex	-2.072	0.759	-2.729	0.007
Age (years)	0.045	0.053	0.848	0.397

Table B32: Outcome VO2peak

	Coefficient β	Std. Error	t-Value	p-Value
ALA	-0.884	1.293	-0.684	0.495
EPA	1.698	0.459	3.703	<0.001
DHA	-0.384	0.177	-2.178	0.030
Sex	2.201	0.311	7.071	<0.001
Age (years)	-0.098	0.022	-4.511	<0.001

Table B33: Outcome Follow-Up Systolic blood pressure, mm Hg

	Coefficient β	Std. Error	t-Value	p-Value
ALA	0.871	7.259	0.120	0.905
EPA	2.015	2.571	0.784	0.434
DHA	-1.045	0.994	-1.051	0.294
Sex	4.358	1.733	2.515	0.012
Age (years)	-0.134	0.120	-1.118	0.264

Table B34: Outcome Follow-Up Diastolic blood pressure, mm Hg

	Coefficient β	Std. Error	t-Value	p-Value
ALA	-3.614	4.341	-0.832	0.406
EPA	-0.611	1.538	-0.397	0.691
DHA	-0.280	0.594	-0.471	0.638
Sex	0.641	1.036	0.619	0.536
Age (years)	-0.413	0.072	-5.759	<0.001

Table B35: Outcome Follow-Up Heart rate, min

	Coefficient β	Std. Error	t-Value	p-Value
ALA	-1.167	4.803	-0.243	0.808
EPA	-0.984	1.701	-0.578	0.563
DHA	-0.375	0.658	-0.571	0.568
Sex	0.080	1.147	0.070	0.944
Age (years)	-0.053	0.079	-0.672	0.502

Table B36: Outcome Follow-Up 6 MWT, (Distance covered) meters

	Coefficient β	Std. Error	t-Value	p-Value
ALA	23.908	38.016	0.629	0.53
EPA	11.563	13.489	0.857	0.392
DHA	18.123	5.259	3.446	0.001
Sex	33.828	9.191	3.681	<0.001
Age (years)	-5.220	0.641	-8.147	<0.001

Table B37: Outcome Follow-Up LDL-C (mg/dl)

	Coefficient β	Std. Error	t-Value	p-Value
ALA	-22.815	17.955	-1.271	0.205
EPA	-3.360	6.356	-0.529	0.597
DHA	1.243	2.462	0.505	0.614
Sex	-12.724	4.302	-2.957	0.003
Age (years)	-0.291	0.297	-0.981	0.327

Table B38: Outcome Follow-Up ASAT

	Coefficient β	Std. Error	t-Value	p-Value
ALA	3.269	3.945	0.829	0.408
EPA	-0.141	1.397	-0.101	0.920
DHA	0.104	0.541	0.192	0.848
Sex	2.736	0.944	2.900	0.004
Age (years)	-0.160	0.065	-2.438	0.015

Table B39: Outcome Follow-Up ALAT

	Coefficient β	Std. Error	t-Value	p-Value
ALA	2.247	5.670	0.396	0.692
EPA	-0.075	2.002	-0.037	0.970
DHA	-0.061	0.775	-0.079	0.937
Sex	3.883	1.353	2.869	0.004
Age (years)	-0.531	0.094	-5.662	<0.001

Table B40: Outcome Follow-Up GGT

	Coefficient β	Std. Error	t-Value	p-Value
ALA	3.010	16.099	0.187	0.852
EPA	5.948	5.722	1.039	0.299
DHA	-2.338	2.215	-1.056	0.292
Sex	7.686	3.852	1.996	0.047
Age (years)	-0.542	0.267	-2.030	0.043

Table B41: Outcome Follow-Up LV ejection fraction, %

	Coefficient β	Std. Error	t-Value	p-Value
ALA	0.933	3.412	0.273	0.785
EPA	-1.310	1.208	-1.084	0.279
DHA	1.393	0.465	2.995	0.003
Sex	-1.902	0.814	-2.336	0.020
Age (years)	0.033	0.056	0.592	0.554

Table B42: Outcome Follow-Up VO2peak

	Coefficient β	Std. Error	t-Value	p-Value
ALA	0.927	1.865	0.497	0.620
EPA	1.439	0.654	2.201	0.028
DHA	0.090	0.255	0.352	0.725
Sex	2.522	0.442	5.701	<0.001
Age (years)	-0.137	0.031	-4.456	<0.001

Table C1: Male Subgroup, Outcome HbA1c

	Coefficient β	Std. Error	t-Value	p-Value
Omega-3 Index (%)	-0.065	0.04	-1.654	0.100
ALA	-0.253	0.51	-0.495	0.621

Table C2: Male Subgroup, Outcome TG/HDL-C ratio

	Coefficient β	Std. Error	t-Value	p-Value
Omega-3 Index (%)	-0.458	0.136	-3.381	0.001
ALA	6.769	1.779	3.805	<0.001

Table C3: Male Subgroup, Outcome Triglycerides (mg/dl)

	Coefficient β	Std. Error	t-Value	p-Value
Omega-3 Index (%)	-17.675	4.832	-3.658	<0.001
ALA	238.157	63.421	3.755	<0.001

Table C4: Male Subgroup, Outcome Non-HDL-C (mg/dl)

	Coefficient β	Std. Error	t-Value	p-Value
Omega-3 Index (%)	-6.138	2.008	-3.057	0.003
ALA	9.286	26.470	0.351	0.726

Table C5: Male Subgroup, Outcome Body mass index

	Coefficient β	Std. Error	t-Value	p-Value
Omega-3 Index (%)	-0.135	0.146	-0.930	0.353
ALA	-0.928	1.926	-0.482	0.630

Table C6: Male Subgroup, Outcome Waist Circumference, (cm)

	Coefficient β	Std. Error	t-Value	p-Value
Omega-3 Index (%)	-0.313	0.399	-0.786	0.433
ALA	4.133	5.276	0.783	0.434

Table C7: Male Subgroup, Outcome Waist-to-height ratio

	Coefficient β	Std. Error	t-Value	p-Value
Omega-3 Index (%)	-0.003	0.003	-0.959	0.339
ALA	-0.021	0.036	-0.564	0.573

Table C8: Male Subgroup, Outcome E/e'

	Coefficient β	Std. Error	t-Value	p-Value
Omega-3 Index (%)	-0.069	0.071	-0.966	0.335
ALA	1.016	0.940	1.080	0.281

Table C9: Male Subgroup, Outcome E/A velocity ratio

	Coefficient β	Std. Error	t-Value	p-Value
Omega-3 Index (%)	0.005	0.015	0.340	0.734
ALA	-0.142	0.205	-0.692	0.490

Table C10: Male Subgroup, Outcome Deceleration time, ms

	Coefficient β	Std. Error	t-Value	p-Value
Omega-3 Index (%)	-0.313	2.963	-0.106	0.916
ALA	6.479	39.206	0.165	0.869

Table C11: Male Subgroup, Outcome NT-proBNP, ng/L

	Coefficient β	Std. Error	t-Value	p-Value
Omega-3 Index (%)	0.030	0.021	1.417	0.158
ALA	0.415	0.275	1.508	0.133

Table C12: Male Subgroup, Outcome Follow-Up HbA1c

	Coefficient β	Std. Error	t-Value	p-Value
Omega-3 Index (%)	-0.011	0.058	-0.196	0.845
ALA	0.702	0.763	0.919	0.359

Table C13: Male Subgroup, Outcome Follow-Up TG/HDL-C ratio

	Coefficient β	Std. Error	t-Value	p-Value
Omega-3 Index (%)	-0.310	0.252	-1.230	0.220
ALA	6.238	3.309	1.885	0.061

Table C14: Male Subgroup, Outcome Follow-Up Triglycerides (mg/dl)

	Coefficient β	Std. Error	t-Value	p-Value
Omega-3 Index (%)	-9.299	6.880	-1.352	0.178
ALA	145.384	90.229	1.611	0.109

Table C15: Male Subgroup, Outcome Follow-Up Non-HDL-C (mg/dl)

	Coefficient β	Std. Error	t-Value	p-Value
Omega-3 Index (%)	-6.764	2.091	-3.235	0.001
ALA	-7.254	27.422	-0.265	0.792

Table C16: Male Subgroup, Outcome Follow-Up Body mass index

	Coefficient β	Std. Error	t-Value	p-Value
Omega-3 Index (%)	-0.184	0.167	-1.105	0.271
ALA	-0.633	2.203	-0.287	0.774

Table C17: Male Subgroup, Outcome Follow-Up Waist Circumference, (cm)

	Coefficient β	Std. Error	t-Value	p-Value
Omega-3 Index (%)	-0.443	0.462	-0.959	0.339
ALA	0.140	6.092	0.023	0.982

Table C18: Male Subgroup, Outcome Follow-Up Waist-to-height ratio

	Coefficient β	Std. Error	t-Value	p-Value
Omega-3 Index (%)	-0.002	0.003	-0.800	0.425
ALA	0.011	0.033	0.317	0.752

Table C19: Male Subgroup, Outcome Follow-Up E/e'

	Coefficient β	Std. Error	t-Value	p-Value
Omega-3 Index (%)	-0.157	0.075	-2.082	0.039
ALA	0.873	0.998	0.874	0.383

Table C20: Male Subgroup, Outcome Follow-Up E/A velocity ratio

	Coefficient β	Std. Error	t-Value	p-Value
Omega-3 Index (%)	-0.007	0.015	-0.465	0.642
ALA	-0.013	0.196	-0.069	0.945

Table C21: Male Subgroup, Outcome Follow-Up Deceleration time, ms

	Coefficient β	Std. Error	t-Value	p-Value
Omega-3 Index (%)	5.689	3.077	1.849	0.066
ALA	7.081	40.752	0.174	0.862

Table C22: Male Subgroup, Outcome Follow-Up NT-proBNP, ng/L

	Coefficient β	Std. Error	t-Value	p-Value
Omega-3 Index (%)	0.051	0.055	0.930	0.354
ALA	0.974	0.704	1.384	0.168

Table C23: Male Subgroup, Outcome Systolic blood pressure, mm Hg

	Coefficient β	Std. Error	t-Value	p-Value
Omega-3 Index (%)	0.035	0.828	0.042	0.966
ALA	-0.933	10.951	-0.085	0.932

Table C24: Male Subgroup, Outcome Diastolic blood pressure, mm Hg

	Coefficient β	Std. Error	t-Value	p-Value
Omega-3 Index (%)	-0.721	0.513	-1.407	0.161
ALA	-9.134	6.787	-1.346	0.180

Table C25: Male Subgroup, Outcome Heart rate, min

	Coefficient β	Std. Error	t-Value	p-Value
Omega-3 Index (%)	-1.205	0.495	-2.432	0.016
ALA	2.890	6.555	0.441	0.660

Table C26: Male Subgroup, Outcome 6 MWT, (Distance covered) meters

	Coefficient β	Std. Error	t-Value	p-Value
Omega-3 Index (%)	5.233	3.231	1.620	0.107
ALA	-13.283	42.749	-0.311	0.756

Table C27: Male Subgroup, Outcome LDL-C (mg/dl)

	Coefficient β	Std. Error	t-Value	p-Value
Omega-3 Index (%)	-3.960	1.826	-2.168	0.031
ALA	-3.003	23.911	-0.126	0.900

Table C28: Male Subgroup, Outcome ASAT

	Coefficient β	Std. Error	t-Value	p-Value
Omega-3 Index (%)	-0.430	0.479	-0.898	0.371
ALA	-0.911	6.320	-0.144	0.886

Table C29: Male Subgroup, Outcome ALAT

	Coefficient β	Std. Error	t-Value	p-Value
Omega-3 Index (%)	-0.523	0.757	-0.691	0.490
ALA	-11.092	9.983	-1.111	0.268

Table C30: Male Subgroup, Outcome GGT

	Coefficient β	Std. Error	t-Value	p-Value
Omega-3 Index (%)	-4.165	1.887	-2.207	0.029
ALA	-10.301	24.878	-0.414	0.679

Table C31: Male Subgroup, Outcome LV ejection fraction, %

	Coefficient β	Std. Error	t-Value	p-Value
Omega-3 Index (%)	0.570	0.347	1.642	0.102
ALA	-0.102	4.595	-0.022	0.982

Table C32: Male Subgroup, Outcome VO2peak

	Coefficient β	Std. Error	t-Value	p-Value
Omega-3 Index (%)	-0.040	0.144	-0.277	0.782
ALA	1.277	1.912	0.668	0.505

Table C33: Male Subgroup, Outcome Follow-Up Systolic blood pressure, mm Hg

	Coefficient β	Std. Error	t-Value	p-Value
Omega-3 Index (%)	-0.530	0.785	-0.675	0.500
ALA	4.248	10.365	0.410	0.682

Table C34: Male Subgroup, Outcome Follow-Up Diastolic blood pressure, mm Hg

	Coefficient β	Std. Error	t-Value	p-Value
Omega-3 Index (%)	-1.295	0.529	-2.448	0.015
ALA	-3.993	6.981	-0.572	0.568

Table C35: Male Subgroup, Outcome Follow-Up Heart rate, min

	Coefficient β	Std. Error	t-Value	p-Value
Omega-3 Index (%)	-1.260	0.551	-2.285	0.023
ALA	4.352	7.275	0.598	0.550

Table C36: Male Subgroup, Outcome Follow-Up 6 MWT, (Distance covered) meters

	Coefficient β	Std. Error	t-Value	p-Value
Omega-3 Index (%)	9.098	4.453	2.043	0.043
ALA	-65.512	59.154	-1.107	0.270

Table C37: Male Subgroup, Outcome Follow-Up LDL-C (mg/dl)

	Coefficient β	Std. Error	t-Value	p-Value
Omega-3 Index (%)	-4.383	1.868	-2.347	0.020
ALA	-21.488	24.492	-0.877	0.382

Table C38: Male Subgroup, Outcome Follow-Up ASAT

	Coefficient β	Std. Error	t-Value	p-Value
Omega-3 Index (%)	-0.276	0.458	-0.604	0.547
ALA	1.446	6.003	0.241	0.810

Table C39: Male Subgroup, Outcome Follow-Up ALAT

	Coefficient β	Std. Error	t-Value	p-Value
Omega-3 Index (%)	-0.742	0.723	-1.027	0.306
ALA	-6.997	9.480	-0.738	0.461

Table C40: Male Subgroup, Outcome Follow-Up GGT

	Coefficient β	Std. Error	t-Value	p-Value
Omega-3 Index (%)	-1.965	1.876	-1.048	0.296
ALA	0.915	24.600	0.037	0.970

Table C41: Male Subgroup, Outcome Follow-Up LV ejection fraction, %

	Coefficient β	Std. Error	t-Value	p-Value
Omega-3 Index (%)	0.220	0.396	0.556	0.579
ALA	6.286	5.239	1.200	0.232

Table C42: Male Subgroup, Outcome Follow-Up VO2peak

	Coefficient β	Std. Error	t-Value	p-Value
Omega-3 Index (%)	0.192	0.220	0.872	0.384
ALA	2.423	2.905	0.834	0.405

Table D1: Male Subgroup, Outcome HbA1c

	Coefficient β	Std. Error	t-Value	p-Value
ALA	-0.407	0.534	-0.762	0.447
EPA	0.089	0.169	0.529	0.597
DHA	-0.122	0.069	-1.778	0.077

Table D2: Male Subgroup, Outcome TG/HDL-C ratio

	Coefficient β	Std. Error	t-Value	p-Value
ALA	7.952	1.844	4.311	<0.001
EPA	-1.701	0.580	-2.934	0.004
DHA	-0.088	0.232	-0.377	0.707

Table D3: Male Subgroup, Outcome Triglycerides (mg/dl)

	Coefficient β	Std. Error	t-Value	p-Value
ALA	265.859	66.230	4.014	<0.001
EPA	-47.174	20.817	-2.266	0.025
DHA	-9.394	8.334	-1.127	0.261

Table D4: Male Subgroup, Outcome Non-HDL-C (mg/dl)

	Coefficient β	Std. Error	t-Value	p-Value
ALA	3.302	27.755	0.119	0.905
EPA	-0.337	8.741	-0.039	0.969
DHA	-8.490	3.478	-2.441	0.016

Table D5: Male Subgroup, Outcome Body mass index

	Coefficient β	Std. Error	t-Value	p-Value
ALA	-0.596	2.021	-0.295	0.768
EPA	-0.485	0.636	-0.762	0.447
DHA	-0.032	0.253	-0.128	0.898

Table D6: Male Subgroup, Outcome Waist Circumference, (cm)

	Coefficient β	Std. Error	t-Value	p-Value
ALA	4.634	5.538	0.837	0.404
EPA	-0.847	1.744	-0.486	0.628
DHA	-0.164	0.693	-0.237	0.813

Table D7: Male Subgroup, Outcome Waist-to-height ratio

	Coefficient β	Std. Error	t-Value	p-Value
ALA	-0.012	0.038	-0.310	0.757
EPA	-0.012	0.012	-0.977	0.330
DHA	0.000	0.005	0.024	0.981

Table D8: Male Subgroup, Outcome E/e'

	Coefficient β	Std. Error	t-Value	p-Value
ALA	1.276	0.985	1.295	0.197
EPA	-0.340	0.310	-1.096	0.274
DHA	0.014	0.123	0.114	0.909

Table D9: Male Subgroup, Outcome E/A velocity ratio

	Coefficient β	Std. Error	t-Value	p-Value
ALA	-0.215	0.214	-1.002	0.318
EPA	0.079	0.067	1.180	0.240
DHA	-0.018	0.026	-0.690	0.491

Table D10: Male Subgroup, Outcome Deceleration time, ms

	Coefficient β	Std. Error	t-Value	p-Value
ALA	31.946	40.690	0.785	0.433
EPA	-26.528	12.814	-2.070	0.040
DHA	8.161	5.088	1.604	0.110

Table D11: Male Subgroup, Outcome NT-proBNP, ng/L

	Coefficient β	Std. Error	t-Value	p-Value
ALA	0.374	0.289	1.296	0.197
EPA	0.073	0.091	0.806	0.421
DHA	0.018	0.037	0.489	0.626

Table D12: Male Subgroup, Outcome Follow-Up HbA1c

	Coefficient β	Std. Error	t-Value	p-Value
ALA	0.802	0.810	0.991	0.323
EPA	-0.101	0.243	-0.417	0.677
DHA	0.017	0.099	0.173	0.863

Table D13: Male Subgroup, Outcome Follow-Up TG/HDL-C ratio

	Coefficient β	Std. Error	t-Value	p-Value
ALA	6.106	3.510	1.740	0.084
EPA	-0.210	1.054	-0.199	0.842
DHA	-0.367	0.429	-0.857	0.393

Table D14: Male Subgroup, Outcome Follow-Up Triglycerides (mg/dl)

	Coefficient β	Std. Error	t-Value	p-Value
ALA	131.188	95.661	1.371	0.172
EPA	2.807	28.718	0.098	0.922
DHA	-13.989	11.680	-1.198	0.233

Table D15: Male Subgroup, Outcome Follow-Up Non-HDL-C (mg/dl)

	Coefficient β	Std. Error	t-Value	p-Value
ALA	-3.400	29.076	-0.117	0.907
EPA	-10.588	8.729	-1.213	0.227
DHA	-6.028	3.550	-1.698	0.091

Table D16: Male Subgroup, Outcome Follow-Up Body mass index

	Coefficient β	Std. Error	t-Value	p-Value
ALA	-0.655	2.337	-0.280	0.780
EPA	-0.175	0.704	-0.249	0.804
DHA	-0.201	0.284	-0.709	0.479

Table D17: Male Subgroup, Outcome Follow-Up Waist Circumference, (cm)

	Coefficient β	Std. Error	t-Value	p-Value
ALA	-0.651	6.462	-0.101	0.920
EPA	0.237	1.947	0.122	0.903
DHA	-0.699	0.786	-0.889	0.375

Table D18: Male Subgroup, Outcome Follow-Up Waist-to-height ratio

	Coefficient β	Std. Error	t-Value	p-Value
ALA	0.001	0.035	0.032	0.975
EPA	0.006	0.011	0.591	0.556
DHA	-0.005	0.004	-1.138	0.257

Table D19: Male Subgroup, Outcome Follow-Up E/e'

	Coefficient β	Std. Error	t-Value	p-Value
ALA	0.492	1.055	0.467	0.641
EPA	0.173	0.317	0.546	0.585
DHA	-0.277	0.128	-2.163	0.032

Table D20: Male Subgroup, Outcome Follow-Up E/A velocity ratio

	Coefficient β	Std. Error	t-Value	p-Value
ALA	-0.002	0.208	-0.007	0.994
EPA	-0.018	0.063	-0.285	0.776
DHA	-0.004	0.025	-0.149	0.882

Table D21: Male Subgroup, Outcome Follow-Up Deceleration time, ms

	Coefficient β	Std. Error	t-Value	p-Value
ALA	27.044	42.980	0.629	0.530
EPA	-11.783	12.904	-0.913	0.362
DHA	11.828	5.211	2.270	0.024

Table D22: Male Subgroup, Outcome Follow-Up NT-proBNP, ng/L

	Coefficient β	Std. Error	t-Value	p-Value
ALA	0.832	0.743	1.121	0.264
EPA	0.191	0.231	0.824	0.411
DHA	0.009	0.094	0.095	0.924

Table D23: Male Subgroup, Outcome Systolic blood pressure, mm Hg

	Coefficient β	Std. Error	t-Value	p-Value
ALA	-3.145	11.486	-0.274	0.785
EPA	2.313	3.617	0.639	0.523
DHA	-0.701	1.436	-0.488	0.626

Table D24: Male Subgroup, Outcome Diastolic blood pressure, mm Hg

	Coefficient β	Std. Error	t-Value	p-Value
ALA	-12.135	7.088	-1.712	0.089
EPA	2.324	2.232	1.041	0.299
DHA	-1.764	0.886	-1.990	0.048

Table D25: Male Subgroup, Outcome Heart rate, min

	Coefficient β	Std. Error	t-Value	p-Value
ALA	5.705	6.848	0.833	0.406
EPA	-4.170	2.157	-1.934	0.055
DHA	-0.336	0.856	-0.392	0.695

Table D26: Male Subgroup, Outcome 6 MWT, (Distance covered) meters

	Coefficient β	Std. Error	t-Value	p-Value
ALA	-9.651	44.880	-0.215	0.830
EPA	1.799	14.133	0.127	0.899
DHA	6.747	5.612	1.202	0.231

Table D27: Male Subgroup, Outcome LDL-C (mg/dl)

	Coefficient β	Std. Error	t-Value	p-Value
ALA	-9.222	25.083	-0.368	0.714
EPA	2.120	7.862	0.270	0.788
DHA	-6.266	3.168	-1.978	0.049

Table D28: Male Subgroup, Outcome ASAT

	Coefficient β	Std. Error	t-Value	p-Value
ALA	0.450	6.628	0.068	0.946
EPA	-1.855	2.087	-0.889	0.375
DHA	-0.001	0.830	-0.001	0.999

Table D29: Male Subgroup, Outcome ALAT

	Coefficient β	Std. Error	t-Value	p-Value
ALA	-9.013	10.470	-0.861	0.390
EPA	-2.692	3.297	-0.816	0.415
DHA	0.141	1.312	0.107	0.915

Table D30: Male Subgroup, Outcome GGT

	Coefficient β	Std. Error	t-Value	p-Value
ALA	-16.149	26.083	-0.619	0.537
EPA	1.610	8.214	0.196	0.845
DHA	-6.358	3.268	-1.946	0.053

Table D31: Male Subgroup, Outcome LV ejection fraction, %

	Coefficient β	Std. Error	t-Value	p-Value
ALA	1.817	4.802	0.378	0.706
EPA	-1.371	1.512	-0.907	0.366
DHA	1.243	0.600	2.071	0.040

Table D32: Male Subgroup, Outcome VO2peak

	Coefficient β	Std. Error	t-Value	p-Value
ALA	-0.510	1.959	-0.260	0.795
EPA	1.796	0.617	2.911	0.004
DHA	-0.638	0.245	-2.606	0.010

Table D33: Male Subgroup, Outcome Follow-Up Systolic blood pressure, mm Hg

	Coefficient β	Std. Error	t-Value	p-Value
ALA	0.578	10.967	0.053	0.958
EPA	2.711	3.304	0.820	0.413
DHA	-1.629	1.334	-1.221	0.224

Table D34: Male Subgroup, Outcome Follow-Up Diastolic blood pressure, mm Hg

	Coefficient β	Std. Error	t-Value	p-Value
ALA	-5.317	7.403	-0.718	0.474
EPA	-0.190	2.230	-0.085	0.932
DHA	-1.755	0.900	-1.949	0.053

Table D35: Male Subgroup, Outcome Follow-Up Heart rate, min

	Coefficient β	Std. Error	t-Value	p-Value
ALA	3.230	7.716	0.419	0.676
EPA	-0.333	2.325	-0.143	0.886
DHA	-1.659	0.939	-1.767	0.079

Table D36: Male Subgroup, Outcome Follow-Up 6 MWT, (Distance covered) meters

	Coefficient β	Std. Error	t-Value	p-Value
ALA	-45.008	62.430	-0.721	0.472
EPA	-9.012	18.765	-0.480	0.632
DHA	15.722	7.584	2.073	0.040

Table D37: Male Subgroup, Outcome Follow-Up LDL-C (mg/dl)

	Coefficient β	Std. Error	t-Value	p-Value
ALA	-10.568	25.857	-0.409	0.683
EPA	-14.364	7.763	-1.850	0.066
DHA	-1.442	3.157	-0.457	0.648

Table D38: Male Subgroup, Outcome Follow-Up ASAT

	Coefficient β	Std. Error	t-Value	p-Value
ALA	4.224	6.335	0.667	0.506
EPA	-2.766	1.902	-1.455	0.148
DHA	0.520	0.773	0.673	0.502

Table D39: Male Subgroup, Outcome Follow-Up ALAT

	Coefficient β	Std. Error	t-Value	p-Value
ALA	-5.142	10.048	-0.512	0.609
EPA	-2.438	3.016	-0.808	0.420
DHA	-0.242	1.227	-0.198	0.844

Table D40: Male Subgroup, Outcome Follow-Up GGT

	Coefficient β	Std. Error	t-Value	p-Value
ALA	-2.765	26.083	-0.106	0.916
EPA	1.198	7.830	0.153	0.879
DHA	-3.155	3.185	-0.991	0.323

Table D41: Male Subgroup, Outcome Follow-Up LV ejection fraction, %

	Coefficient β	Std. Error	t-Value	p-Value
ALA	6.914	5.556	1.244	0.215
EPA	-0.327	1.668	-0.196	0.845
DHA	0.415	0.674	0.617	0.538

Table D42: Male Subgroup, Outcome Follow-Up VO2peak

	Coefficient β	Std. Error	t-Value	p-Value
ALA	0.966	3.066	0.315	0.753
EPA	1.501	0.927	1.620	0.107
DHA	-0.232	0.380	-0.611	0.542

Table E1: Female Subgroup, Outcome HbA1c

	Coefficient β	Std. Error	t-Value	p-Value
Omega-3 Index (%)	-0.054	0.027	-2.011	0.046
ALA	0.024	0.335	0.072	0.943

Table E2: Female Subgroup, Outcome TG/HDL-C ratio

	Coefficient β	Std. Error	t-Value	p-Value
Omega-3 Index (%)	-0.155	0.103	-1.502	0.135
ALA	4.042	1.351	2.991	0.003

Table E3: Female Subgroup, Outcome Triglycerides (mg/dl)

	Coefficient β	Std. Error	t-Value	p-Value
Omega-3 Index (%)	-4.132	3.969	-1.041	0.299
ALA	147.024	51.832	2.837	0.005

Table E4: Female Subgroup, Outcome Non-HDL-C (mg/dl)

	Coefficient β	Std. Error	t-Value	p-Value
Omega-3 Index (%)	0.333	2.071	0.161	0.872
ALA	5.121	26.030	0.197	0.844

Table E5: Female Subgroup, Outcome Body mass index

	Coefficient β	Std. Error	t-Value	p-Value
Omega-3 Index (%)	-0.471	0.161	-2.926	0.004
ALA	-1.655	2.042	-0.810	0.419

Table E6: Female Subgroup, Outcome Waist Circumference, (cm)

	Coefficient β	Std. Error	t-Value	p-Value
Omega-3 Index (%)	-1.417	0.427	-3.320	0.001
ALA	1.118	5.429	0.206	0.837

Table E7: Female Subgroup, Outcome Waist-to-height ratio

	Coefficient β	Std. Error	t-Value	p-Value
Omega-3 Index (%)	-0.008	0.003	-3.062	0.002
ALA	0.004	0.033	0.112	0.911

Table E8: Female Subgroup, Outcome E/e'

	Coefficient β	Std. Error	t-Value	p-Value
Omega-3 Index (%)	-0.014	0.064	-0.223	0.824
ALA	0.419	0.813	0.516	0.606

Table E9: Female Subgroup, Outcome E/A velocity ratio

	Coefficient β	Std. Error	t-Value	p-Value
Omega-3 Index (%)	-0.016	0.014	-1.146	0.253
ALA	0.249	0.182	1.367	0.173

Table E10: Female Subgroup, Outcome Deceleration time, ms

	Coefficient β	Std. Error	t-Value	p-Value
Omega-3 Index (%)	1.730	2.503	0.691	0.490
ALA	-19.584	31.739	-0.617	0.538

Table E11: Female Subgroup, Outcome NT-proBNP, ng/L

	Coefficient β	Std. Error	t-Value	p-Value
Omega-3 Index (%)	0.027	0.018	1.490	0.138
ALA	0.722	0.225	3.212	0.002

Table E12: Female Subgroup, Outcome Follow-Up HbA1c

	Coefficient β	Std. Error	t-Value	p-Value
Omega-3 Index (%)	-0.038	0.024	-1.559	0.121
ALA	0.064	0.297	0.216	0.829

Table E13: Female Subgroup, Outcome Follow-Up TG/HDL-C ratio

	Coefficient β	Std. Error	t-Value	p-Value
Omega-3 Index (%)	-0.096	0.085	-1.123	0.263
ALA	1.069	1.054	1.015	0.311

Table E14: Female Subgroup, Outcome Follow-Up Triglycerides (mg/dl)

	Coefficient β	Std. Error	t-Value	p-Value
Omega-3 Index (%)	0.802	3.565	0.225	0.822
ALA	26.607	43.907	0.606	0.545

Table E15: Female Subgroup, Outcome Follow-Up Non-HDL-C (mg/dl)

	Coefficient β	Std. Error	t-Value	p-Value
Omega-3 Index (%)	2.399	2.123	1.13	0.260
ALA	-24.685	26.261	-0.94	0.348

Table E16: Female Subgroup, Outcome Follow-Up Body mass index

	Coefficient β	Std. Error	t-Value	p-Value
Omega-3 Index (%)	-0.452	0.173	-2.618	0.010
ALA	-1.302	2.127	-0.612	0.541

Table E17: Female Subgroup, Outcome Follow-Up Waist Circumference, (cm)

	Coefficient β	Std. Error	t-Value	p-Value
Omega-3 Index (%)	-1.644	0.449	-3.663	<0.001
ALA	-1.247	5.431	-0.230	0.819

Table E18: Female Subgroup, Outcome Follow-Up Waist-to-height ratio

	Coefficient β	Std. Error	t-Value	p-Value
Omega-3 Index (%)	-0.010	0.003	-3.350	0.001
ALA	-0.046	0.035	-1.315	0.190

Table E19: Female Subgroup, Outcome Follow-Up E/e'

	Coefficient β	Std. Error	t-Value	p-Value
Omega-3 Index (%)	-0.063	0.078	-0.815	0.416
ALA	1.298	0.956	1.358	0.176

Table E20: Female Subgroup, Outcome Follow-Up E/A velocity ratio

	Coefficient β	Std. Error	t-Value	p-Value
Omega-3 Index (%)	-0.009	0.016	-0.568	0.570
ALA	0.128	0.198	0.647	0.519

Table E21: Female Subgroup, Outcome Follow-Up Deceleration time, ms

	Coefficient β	Std. Error	t-Value	p-Value
Omega-3 Index (%)	4.848	2.760	1.757	0.081
ALA	8.072	34.002	0.237	0.813

Table E22: Female Subgroup, Outcome Follow-Up NT-proBNP, ng/L

	Coefficient β	Std. Error	t-Value	p-Value
Omega-3 Index (%)	0.004	0.042	0.088	0.930
ALA	2.033	0.509	3.996	<0.001

Table E23: Female Subgroup, Outcome Systolic blood pressure, mm Hg

	Coefficient β	Std. Error	t-Value	p-Value
Omega-3 Index (%)	-0.557	0.752	-0.740	0.460
ALA	8.719	9.534	0.914	0.362

Table E24: Female Subgroup, Outcome Diastolic blood pressure, mm Hg

	Coefficient β	Std. Error	t-Value	p-Value
Omega-3 Index (%)	-0.257	0.429	-0.598	0.550
ALA	-3.582	5.440	-0.658	0.511

Table E25: Female Subgroup, Outcome Heart rate, min

	Coefficient β	Std. Error	t-Value	p-Value
Omega-3 Index (%)	1.098	0.484	2.269	0.024
ALA	3.611	6.139	0.588	0.557

Table E26: Female Subgroup, Outcome 6 MWT, (Distance covered) meters

	Coefficient β	Std. Error	t-Value	p-Value
Omega-3 Index (%)	6.791	3.862	1.758	0.080
ALA	-33.443	50.533	-0.662	0.509

Table E27: Female Subgroup, Outcome LDL-C (mg/dl)

	Coefficient β	Std. Error	t-Value	p-Value
Omega-3 Index (%)	2.397	1.833	1.308	0.192
ALA	-5.499	23.209	-0.237	0.813

Table E28: Female Subgroup, Outcome ASAT

	Coefficient β	Std. Error	t-Value	p-Value
Omega-3 Index (%)	-0.143	0.355	-0.401	0.689
ALA	-1.241	4.499	-0.276	0.783

Table E29: Female Subgroup, Outcome ALAT

	Coefficient β	Std. Error	t-Value	p-Value
Omega-3 Index (%)	-0.643	0.521	-1.235	0.218
ALA	-4.942	6.592	-0.750	0.454

Table E30: Female Subgroup, Outcome GGT

	Coefficient β	Std. Error	t-Value	p-Value
Omega-3 Index (%)	-1.530	1.556	-0.983	0.327
ALA	5.678	19.692	0.288	0.773

Table E31: Female Subgroup, Outcome LV ejection fraction, %

	Coefficient β	Std. Error	t-Value	p-Value
Omega-3 Index (%)	1.451	0.313	4.636	<0.001
ALA	-4.859	3.968	-1.225	0.222

Table E32: Female Subgroup, Outcome VO2peak

	Coefficient β	Std. Error	t-Value	p-Value
Omega-3 Index (%)	0.002	0.136	0.016	0.987
ALA	-2.359	1.731	-1.363	0.174

Table E33: Female Subgroup, Outcome Follow-Up Systolic blood pressure, mm Hg

	Coefficient β	Std. Error	t-Value	p-Value
Omega-3 Index (%)	-0.388	0.754	-0.515	0.607
ALA	-0.708	9.208	-0.077	0.939

Table E34: Female Subgroup, Outcome Follow-Up Diastolic blood pressure, mm Hg

	Coefficient β	Std. Error	t-Value	p-Value
Omega-3 Index (%)	-0.341	0.430	-0.793	0.429
ALA	-10.221	5.248	-1.947	0.053

Table E35: Female Subgroup, Outcome Follow-Up Heart rate, min

	Coefficient β	Std. Error	t-Value	p-Value
Omega-3 Index (%)	0.023	0.464	0.049	0.961
ALA	-5.557	5.666	-0.981	0.328

Table E36: Female Subgroup, Outcome Follow-Up 6 MWT, (Distance covered) meters

	Coefficient β	Std. Error	t-Value	p-Value
Omega-3 Index (%)	11.523	4.367	2.639	0.009
ALA	-8.661	51.745	-0.167	0.867

Table E37: Female Subgroup, Outcome Follow-Up LDL-C (mg/dl)

	Coefficient β	Std. Error	t-Value	p-Value
Omega-3 Index (%)	3.693	1.880	1.964	0.051
ALA	-23.533	23.117	-1.018	0.310

Table E38: Female Subgroup, Outcome Follow-Up ASAT

	Coefficient β	Std. Error	t-Value	p-Value
Omega-3 Index (%)	0.008	0.389	0.022	0.983
ALA	1.816	4.755	0.382	0.703

Table E39: Female Subgroup, Outcome Follow-Up ALAT

	Coefficient β	Std. Error	t-Value	p-Value
Omega-3 Index (%)	-0.467	0.532	-0.877	0.382
ALA	0.046	6.540	0.007	0.994

Table E40: Female Subgroup, Outcome Follow-Up GGT

	Coefficient β	Std. Error	t-Value	p-Value
Omega-3 Index (%)	0.131	1.575	0.083	0.934
ALA	6.673	19.232	0.347	0.729

Table E41: Female Subgroup, Outcome Follow-Up LV ejection fraction, %

	Coefficient β	Std. Error	t-Value	p-Value
Omega-3 Index (%)	1.111	0.324	3.428	0.001
ALA	-4.594	3.992	-1.151	0.251

Table E42: Female Subgroup, Outcome Follow-Up VO2peak

	Coefficient β	Std. Error	t-Value	p-Value
Omega-3 Index (%)	0.266	0.181	1.471	0.143
ALA	-1.484	2.258	-0.658	0.512

Table F1: Female Subgroup, Outcome HbA1c

	Coefficient β	Std. Error	t-Value	p-Value
ALA	-0.072	0.345	-0.208	0.836
EPA	0.111	0.145	0.764	0.446
DHA	-0.109	0.052	-2.092	0.038

Table F2: Female Subgroup, Outcome TG/HDL-C ratio

	Coefficient β	Std. Error	t-Value	p-Value
ALA	4.271	1.391	3.071	0.002
EPA	-0.557	0.564	-0.987	0.325
DHA	-0.043	0.202	-0.215	0.830

Table F3: Female Subgroup, Outcome Triglycerides (mg/dl)

	Coefficient β	Std. Error	t-Value	p-Value
ALA	144.113	53.479	2.695	0.008
EPA	0.531	21.776	0.024	0.981
DHA	-5.875	7.791	-0.754	0.452

Table F4: Female Subgroup, Outcome Non-HDL-C (mg/dl)

	Coefficient β	Std. Error	t-Value	p-Value
ALA	-4.220	26.627	-0.159	0.874
EPA	17.302	11.122	1.556	0.121
DHA	-4.868	4.006	-1.215	0.226

Table F5: Female Subgroup, Outcome Body mass index

	Coefficient β	Std. Error	t-Value	p-Value
ALA	-0.598	2.075	-0.288	0.774
EPA	-2.424	0.866	-2.798	0.006
DHA	0.088	0.309	0.285	0.776

Table F6: Female Subgroup, Outcome Waist Circumference, (cm)

	Coefficient β	Std. Error	t-Value	p-Value
ALA	3.676	5.534	0.664	0.507
EPA	-6.094	2.305	-2.644	0.009
DHA	-0.100	0.821	-0.122	0.903

Table F7: Female Subgroup, Outcome Waist-to-height ratio

	Coefficient β	Std. Error	t-Value	p-Value
ALA	0.022	0.034	0.659	0.511
EPA	-0.042	0.014	-2.997	0.003
DHA	0.002	0.005	0.352	0.725

Table F8: Female Subgroup, Outcome E/e'

	Coefficient β	Std. Error	t-Value	p-Value
ALA	0.605	0.834	0.725	0.469
EPA	-0.353	0.348	-1.014	0.312
DHA	0.088	0.124	0.706	0.481

Table F9: Female Subgroup, Outcome E/A velocity ratio

	Coefficient β	Std. Error	t-Value	p-Value
ALA	0.228	0.187	1.220	0.224
EPA	0.022	0.076	0.289	0.773
DHA	-0.029	0.027	-1.059	0.291

Table F10: Female Subgroup, Outcome Deceleration time, ms

	Coefficient β	Std. Error	t-Value	p-Value
ALA	-19.628	32.650	-0.601	0.548
EPA	1.911	13.632	0.140	0.889
DHA	1.805	4.860	0.371	0.711

Table F11: Female Subgroup, Outcome NT-proBNP, ng/L

	Coefficient β	Std. Error	t-Value	p-Value
ALA	0.719	0.231	3.107	0.002
EPA	0.035	0.096	0.370	0.712
DHA	0.026	0.035	0.745	0.457

Table F12: Female Subgroup, Outcome Follow-Up HbA1c

	Coefficient β	Std. Error	t-Value	p-Value
ALA	0.027	0.307	0.089	0.929
EPA	0.026	0.136	0.191	0.849
DHA	-0.060	0.048	-1.249	0.213

Table F13: Female Subgroup, Outcome Follow-Up TG/HDL-C ratio

	Coefficient β	Std. Error	t-Value	p-Value
ALA	1.359	1.083	1.255	0.211
EPA	-0.644	0.484	-1.330	0.185
DHA	0.063	0.170	0.372	0.711

Table F14: Female Subgroup, Outcome Follow-Up Triglycerides (mg/dl)

	Coefficient β	Std. Error	t-Value	p-Value
ALA	32.837	45.336	0.724	0.470
EPA	-10.417	20.236	-0.515	0.607
DHA	4.256	7.102	0.599	0.550

Table F15: Female Subgroup, Outcome Follow-Up Non-HDL-C (mg/dl)

	Coefficient β	Std. Error	t-Value	p-Value
ALA	-30.736	27.070	-1.135	0.258
EPA	13.472	12.016	1.121	0.264
DHA	-0.747	4.198	-0.178	0.859

Table F16: Female Subgroup, Outcome Follow-Up Body mass index

	Coefficient β	Std. Error	t-Value	p-Value
ALA	-0.425	2.188	-0.194	0.846
EPA	-2.010	0.970	-2.073	0.039
DHA	-0.018	0.339	-0.054	0.957

Table F17: Female Subgroup, Outcome Follow-Up Waist Circumference, (cm)

	Coefficient β	Std. Error	t-Value	p-Value
ALA	1.055	5.594	0.189	0.851
EPA	-5.658	2.473	-2.288	0.023
DHA	-0.544	0.878	-0.620	0.536

Table F18: Female Subgroup, Outcome Follow-Up Waist-to-height ratio

	Coefficient β	Std. Error	t-Value	p-Value
ALA	-0.034	0.036	-0.929	0.354
EPA	-0.031	0.016	-1.971	0.050
DHA	-0.004	0.006	-0.666	0.506

Table F19: Female Subgroup, Outcome Follow-Up E/e'

	Coefficient β	Std. Error	t-Value	p-Value
ALA	1.251	0.989	1.265	0.207
EPA	0.015	0.438	0.034	0.973
DHA	-0.091	0.153	-0.596	0.552

Table F20: Female Subgroup, Outcome Follow-Up E/A velocity ratio

	Coefficient β	Std. Error	t-Value	p-Value
ALA	0.085	0.204	0.416	0.678
EPA	0.069	0.089	0.771	0.442
DHA	-0.033	0.031	-1.057	0.292

Table F21: Female Subgroup, Outcome Follow-Up Deceleration time, ms

	Coefficient β	Std. Error	t-Value	p-Value
ALA	0.384	35.129	0.011	0.991
EPA	18.568	15.571	1.192	0.235
DHA	1.095	5.440	0.201	0.841

Table F22: Female Subgroup, Outcome Follow-Up NT-proBNP, ng/L

	Coefficient β	Std. Error	t-Value	p-Value
ALA	1.972	0.526	3.746	<0.001
EPA	0.111	0.234	0.476	0.634
DHA	-0.029	0.082	-0.346	0.729

Table F23: Female Subgroup, Outcome Systolic blood pressure, mm Hg

	Coefficient β	Std. Error	t-Value	p-Value
ALA	10.053	9.799	1.026	0.306
EPA	-3.020	4.091	-0.738	0.461
DHA	0.152	1.459	0.104	0.917

Table F24: Female Subgroup, Outcome Diastolic blood pressure, mm Hg

	Coefficient β	Std. Error	t-Value	p-Value
ALA	-2.804	5.591	-0.501	0.617
EPA	-1.690	2.335	-0.724	0.470
DHA	0.160	0.832	0.193	0.847

Table F25: Female Subgroup, Outcome Heart rate, min

	Coefficient β	Std. Error	t-Value	p-Value
ALA	4.554	6.308	0.722	0.471
EPA	-0.556	2.634	-0.211	0.833
DHA	1.685	0.939	1.794	0.074

Table F26: Female Subgroup, Outcome 6 MWT, (Distance covered) meters

	Coefficient β	Std. Error	t-Value	p-Value
ALA	-43.622	51.993	-0.839	0.402
EPA	24.558	21.037	1.167	0.244
DHA	1.892	7.498	0.252	0.801

Table F27: Female Subgroup, Outcome LDL-C (mg/dl)

	Coefficient β	Std. Error	t-Value	p-Value
ALA	-12.694	23.749	-0.535	0.594
EPA	15.861	9.933	1.597	0.112
DHA	-1.516	3.539	-0.429	0.669

Table F28: Female Subgroup, Outcome ASAT

	Coefficient β	Std. Error	t-Value	p-Value
ALA	-3.010	4.602	-0.654	0.514
EPA	3.028	1.934	1.566	0.119
DHA	-1.126	0.692	-1.627	0.105

Table F29: Female Subgroup, Outcome ALAT

	Coefficient β	Std. Error	t-Value	p-Value
ALA	-6.971	6.761	-1.031	0.304
EPA	2.965	2.841	1.044	0.298
DHA	-1.799	1.017	-1.769	0.078

Table F30: Female Subgroup, Outcome GGT

	Coefficient β	Std. Error	t-Value	p-Value
ALA	-4.345	20.052	-0.217	0.829
EPA	16.394	8.426	1.946	0.053
DHA	-7.144	3.015	-2.369	0.019

Table F31: Female Subgroup, Outcome LV ejection fraction, %

	Coefficient β	Std. Error	t-Value	p-Value
ALA	-4.011	4.073	-0.985	0.326
EPA	-0.010	1.701	-0.006	0.995
DHA	2.005	0.606	3.307	0.001

Table F32: Female Subgroup, Outcome VO2peak

	Coefficient β	Std. Error	t-Value	p-Value
ALA	-3.080	1.767	-1.744	0.083
EPA	1.318	0.738	1.787	0.075
DHA	-0.398	0.263	-1.515	0.131

Table F33: Female Subgroup, Outcome Follow-Up Systolic blood pressure, mm Hg

	Coefficient β	Std. Error	t-Value	p-Value
ALA	-1.322	9.536	-0.139	0.890
EPA	0.650	4.214	0.154	0.878
DHA	-0.731	1.482	-0.493	0.622

Table F34: Female Subgroup, Outcome Follow-Up Diastolic blood pressure, mm Hg

	Coefficient β	Std. Error	t-Value	p-Value
ALA	-9.440	5.431	-1.738	0.084
EPA	-1.709	2.400	-0.712	0.477
DHA	0.047	0.844	0.055	0.956

Table F35: Female Subgroup, Outcome Follow-Up Heart rate, min

	Coefficient β	Std. Error	t-Value	p-Value
ALA	-4.005	5.852	-0.684	0.494
EPA	-2.657	2.586	-1.027	0.306
DHA	0.833	0.909	0.916	0.361

Table F36: Female Subgroup, Outcome Follow-Up 6 MWT, (Distance covered) meters

	Coefficient β	Std. Error	t-Value	p-Value
ALA	-34.453	53.544	-0.643	0.521
EPA	53.299	24.029	2.218	0.028
DHA	-0.293	8.508	-0.034	0.973

Table F37: Female Subgroup, Outcome Follow-Up LDL-C (mg/dl)

	Coefficient β	Std. Error	t-Value	p-Value
ALA	-29.385	23.830	-1.233	0.219
EPA	14.398	10.567	1.363	0.175
DHA	0.741	3.709	0.200	0.842

Table F38: Female Subgroup, Outcome Follow-Up ASAT

	Coefficient β	Std. Error	t-Value	p-Value
ALA	-0.499	4.877	-0.102	0.919
EPA	4.055	2.163	1.875	0.062
DHA	-1.205	0.757	-1.592	0.113

Table F39: Female Subgroup, Outcome Follow-Up ALAT

	Coefficient β	Std. Error	t-Value	p-Value
ALA	-2.566	6.724	-0.382	0.703
EPA	4.087	2.965	1.378	0.170
DHA	-1.867	1.038	-1.799	0.074

Table F40: Female Subgroup, Outcome Follow-Up GGT

	Coefficient β	Std. Error	t-Value	p-Value
ALA	-1.511	19.797	-0.076	0.939
EPA	14.359	8.870	1.619	0.107
DHA	-4.142	3.103	-1.335	0.183

Table F41: Female Subgroup, Outcome Follow-Up LV ejection fraction, %

	Coefficient β	Std. Error	t-Value	p-Value
ALA	-1.840	4.057	-0.453	0.651
EPA	-3.639	1.799	-2.023	0.044
DHA	2.620	0.628	4.169	<0.001

Table F42: Female Subgroup, Outcome Follow-Up VO2peak

	Coefficient β	Std. Error	t-Value	p-Value
ALA	-2.023	2.327	-0.869	0.386
EPA	1.244	1.023	1.216	0.225
DHA	-0.009	0.358	-0.026	0.979
